# Germanene Reformation from Oxidized Germanene on Ag(111)/Ge(111) by Vacuum Annealing

**DOI:** 10.1002/smtd.202400863

**Published:** 2024-09-09

**Authors:** Seiya Suzuki, Daiki Katsube, Masahiro Yano, Yasutaka Tsuda, Tomo‐o Terasawa, Takahiro Ozawa, Katsuyuki Fukutani, Yousoo Kim, Hidehito Asaoka, Junji Yuhara, Akitaka Yoshigoe

**Affiliations:** ^1^ Advanced Science Research Center (ASRC) Japan Atomic Energy Agency (JAEA) 2–4 Shirakata, Tokai‐mura, Naka‐gun Ibaraki 319–1195 Japan; ^2^ Cluster for Pioneering Research RIKEN 2‐1 Hirosawa, Wako Saitama 351‐0198 Japan; ^3^ Materials Sciences Research Center Japan Atomic Energy Agency (JAEA) 1‐1‐1 Kouto, Sayo‐cho, Sayo‐gun Hyogo 679–5148 Japan; ^4^ Institute of Industrial Science The University of Tokyo 4‐6‐1 Komaba Meguro‐ku Tokyo 153–8505 Japan; ^5^ Department of Energy Engineering Nagoya University Furo‐cho, Chikusa‐ku Nagoya Aichi 464–8603 Japan

**Keywords:** Ag(111), desorption, germanene, oxidized germanene, segregation method

## Abstract

For group 14 mono‐elemental 2D materials, such as silicene, germanene, and stanene, oxidation is a severe problem that alters or degrades their physical properties. This study shows that the oxidized germanene on Ag(111)/Ge(111) can be reformed to germanene by simple heating ≈500 °C in a vacuum. The key reaction in reforming germanene is the desorption of GeO and GeO_2_ during heating ≈350 °C. After removing surface oxygen, Ge further segregates to the surface, resulting in the reformation of germanene. The reformed germanene has the same crystal structure, a (7√7 × 7√7) R19.1° supercell with respect to Ag(111), and has equivalent high quality to that of as‐grown germanene on Ag(111)/Ge(111). Even after air oxidation, germanene can be reformed by annealing in a vacuum. On the other hand, the desorption of GeO and GeO_2_ at high temperatures is not suppressed in the O_2_ backfilling atmosphere. This instability of oxidized germanene/Ag(111)/Ge(111) at high temperatures contributes to the ease of germanene reformation without residual oxygen. In other words, the present germanene reformation, as well as the segregation of germanene on Ag(111)/Ge(111), is a highly robust process to synthesize germanene.

## Graphical abstract

Oxidized germanene on Ag(111)/Ge(111) returns to germanene by a simple annealing in a vacuum. The mechanism is that the oxygen in oxidized germanene is desorbed as GeO and GeO_2_ by heating, and the resupplied Ge from the substrate allows the reformation of germanene. Germanene and oxidized germanene on Ag(111)/Ge(111) can be repeatedly used by the developed oxidation and regrowth process.

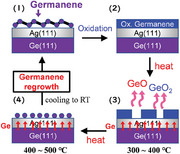

## 1 Introduction

Group 14 mono‐elemental 2D materials beyond graphene, such as silicene,^[^ ^1^ ^]^ germanene,^[^ ^2^ ^]^ stanene,^[^ ^3^ ^]^ and plumbene^[^ ^4^ ^]^ (called group 14 Xenes^[^ ^5^ ^]^) are of great interest to researchers. Theoretical studies of these materials are well ahead of experimental studies and predict outstanding physical properties, such as a tunable bandgap,^[^ ^6^ ^]^ high mobility,^[^ ^7^ ^]^ quantum spin Hall system,^[^ ^8^ ^]^ and 2D topological insulators.^[^ ^9^ ^]^ One of the important properties of these group 14 monolayers is the sizable bandgap induced by the spin‐orbit coupling effect.^[^ ^10^ ^]^ The size of the bandgap increases as the elemental mass number, with theoretically reported values of 1.5 meV for silicene,^[^ ^10^, ^11^ ^]^ 24 meV for germanene,^[^ ^10^, ^11^, ^12^ ^]^ 0.3 eV for stanene,^[^ ^10^, ^13^ ^]^ and 0.7 eV for plumbene,^[^ ^13b^ ^]^ all notably larger than that of graphene (≈50 µeV^[^ ^10^, ^12^, ^14^ ^]^). The larger bandgap allows more robust topological properties to thermal energy. Thermally robust topological properties can lead to the creation of robust topological qubits, which can enable stable quantum computing. Hence, the topological properties of germanene, stanene, and plumbene are expected to be effective above room temperature (RT) and contribute to quantum computing applications.

The growth of these 2D monolayers has already been widely reported. Molecular beam epitaxy (MBE) on surfaces is one of the primary growth methods. The number of growth reports on stanene^[^ ^3^ ^]^ and plumbene^[^ ^4a^ ^]^ have been limited, but germanene is abundant. Various types of surface, such as Ag(111),^[^ ^15^ ^]^ Au(111),^[^ ^2^, ^16^ ^]^ Cu(111),^[^ ^17^ ^]^ Al(111),^[^ ^18^ ^]^ Pt(111),^[^ ^19^ ^]^ Ag_0.9_Al_0.1_(111) alloy,^[^ ^20^ ^]^ MoS_2_,^[^ ^21^ ^]^ and graphite^[^ ^22^ ^]^ are available for the growth of germanene, which may imply the ease of the growth among group 14 monolayers other than graphene. However, no electronic devices from germanene have been achieved. Even including the related group 14 monolayers, there are limited reports for silicene^[^ ^23^ ^]^ and hydrogenated multilayer germanene transistors.^[^ ^24^ ^]^ One of the reasons for the difficulty in fabricating electronic devices is the chemical instability of germanene. Germanene is easily oxidized in air, which is significantly different from graphene^[^ ^25^ ^]^ and transitional metal dichalcogenides monolayers.^[^ ^26^ ^]^ Although the fabrication process for germanene devices has not yet been established, oxidation of germanene can be a serious problem that severely degrades the device's properties. Therefore, it is important to avoid the oxidation of germanene; otherwise, it is necessary to recover germanene from its oxidized state. To protect germanene from oxidation, the growth of germanene at the interface between hexagonal boron nitride/Ag(111) and graphene/Ag(111) has been reported.^[^ ^15b^ ^]^ On the other hand, oxygen can generally be removed chemically, electrochemically, photocatalytically, and thermally. Among the oxygen removal methods, thermal annealing would be suitable because of its high compatibility with dry processes in the semiconductor industry. The removal of surface oxides by thermal methods has been widely reported for various materials, such as graphene oxide,^[^ ^27^ ^]^ Ag nanoparticles,^[^ ^28^ ^]^ stainless steels,^[^ ^29^ ^]^ and TiO_2_.^[^ ^30^ ^]^ However, there are no reports on the thermal annealing of oxidized germanene, nor are there reports on the effects of germanene oxidation to address the oxidation problems of germanene.

Here, we investigated the vacuum annealing effect of oxidized germanene on Ag(111)/Ge(111). The first finding was that oxidized germanene on Ag(111)/Ge(111) can be completely reformed to germanene by simple heating in ultra‐high vacuum (UHV). Germanene was grown by a segregation method,^[^ ^15a^ ^]^ and its structure was confirmed by low energy electron diffraction (LEED) and synchrotron radiation X‐ray photoelectron spectroscopy (SRXPS). For the oxidation of germanene, we used in situ O_2_ backfilling and ex situ air exposure, and both of them resulted in the reformation of germanene after UHV heating. Low temperature scanning tunneling microscopy (LTSTM) revealed that the atomic scale structure of reformed germanene possesses a (7√7 × 7√7) R19.1° supercell with respect to Ag(111), indicating that the crystal structure of reformed germanene is equivalent to that of as‐grown germanene. To elucidate the mechanism of germanene reformation, we investigated the temperature dependence of Ge 3*d* and O 1*s* SRXPS spectra as well as thermal desorption spectroscopy (TDS) of the oxidized germanene. The results revealed that the key reaction in reforming germanene from its oxidized phase is the desorption of GeO and GeO_2_ during heating. The desorption of GeO and GeO_2_ at high temperatures was not suppressed even in the O_2_ backfilling atmosphere. This instability of oxidized germanene/Ag(111)/Ge(111) at high temperatures contributes to the ease of germanene reformation without oxygen contamination, which we expect to be compatible with the dry processes in the device fabrication.

## 2 Results and Discussion

**Figure 1:**
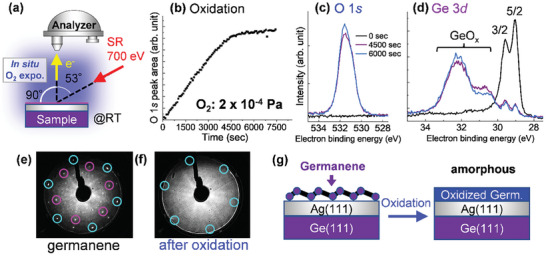
In situ oxidation of germanene by O_2_ backfilling at RT. a) Schematic of in situ SRXPS measurement during oxidation of germanene. b) Evolution of the peak area of O 1*s* with O_2_ exposure time at the 2 × 10^−4^ Pa of oxygen partial pressure. c,d) O 1*s* (c) and Ge 3*d* (d) SRXPS spectra after the O_2_ exposure for 0, 4500, and 6000 sec. e,f) LEED patterns of germanene before (e) and after oxidation (f). The incident beam energy for LEED is 70 eV. The blue and purple circles show (1 × 1) spots of Ag(111) and germanene, respectively. The inner spots correspond to the superstructure between Ag(111) and germanene. g) Schematic of the oxidation of germanene.

**Figure** **1** shows the in situ oxidation and SRXPS of germanene. Figure 1a shows a schematic of the in situ SRXPS measurement. Figure 1b shows the evolution of the O 1*s* peak area with respect to time for an O_2_ exposure of 2 × 10^−4^ Pa. Figure 1c,d show O 1*s* and Ge 3*d* SRXPS spectra after the O_2_ exposure for 0, 4500, and 6000 sec, respectively. The increase in O 1*s* (Figure 1b,c) and the peak shift of Ge 3*d* to higher binding energies (Figure 1d) after the O_2_ exposure clearly indicate that the oxidation of germanene occurred at RT (see also Figure S14, Supporting Information). As can be seen from the transition of the O 1*s* peak area to a plateau in Figure 1b, the oxidation reaction of germanene is nearly saturated at ≈4500 sec of O_2_ exposure, which corresponds to ≈6000 Langmuir (L). The saturation of the oxidation reaction is also confirmed by the appearance of nearly identical spectra of O 1*s* and Ge 3*d* at exposure times of 4500 and 6000 sec, as shown in Figure 1c,d. It should be noted that the oxidation of germanene by O_2_ molecules in the present study is considerably accelerated by the SR and the two Bayard‐Alpert (BA) hot cathode ionization gauges to monitor chamber pressure. While the SR simply accelerated the oxidation rate of germanene by ≈2–4 times (Figure S1, Supporting Information), the two BA gauges altered not only the reaction rate but also the oxidized state of germanene (Figure S2, Supporting Information). The oxidation state of germanene resulting from the SR and the BA gauges is similar to that of germanene oxidized in air (as shown later in Figure 3), indicating that the germanene oxidized in our UHV chamber is close to the naturally stable one in air.

The crystal structure of as‐grown germanene was confirmed by LEED, as shown in Figure 1e. The blue and purple circled spots correspond to the (1 × 1) spots of Ag(111) and germanene, respectively. The inner spots correspond to the superstructure between the Ag(111) surface and germanene, which has been reported previously.^[^ ^15a^ ^]^ Since the LEED patterns of germanene have disappeared by oxidation (Figure 1f), the crystal structure of oxidized germanene is amorphous. Thus, the oxidation of germanene on Ag(111)/Ge(111) can be briefly summarized as shown in Figure 1g.

**Figure 2:**
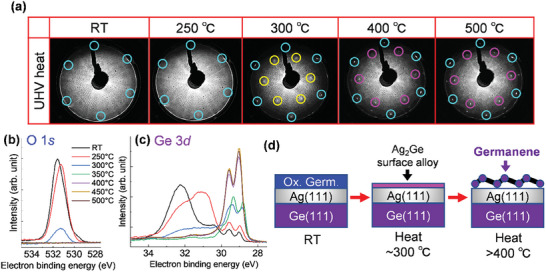
Germanene reformation by UHV heating. a) LEED patterns of oxidized germanene at RT after different heating temperatures. The incident beam energy for LEED is 70 eV. b,c) O 1*s* (b) and Ge 3*d* (c) SRXPS spectra of oxidized germanene at RT after heating at different temperatures. d) Schematic of germanene reformation by UHV heating.

**Figure** **2** shows germanene reformation from oxidized germanene by UHV heating. Figure 2a‐c shows the LEED patterns, O 1*s*, and Ge 3*d* SRXPS spectra of the oxidized germanene after heating at different temperatures, respectively. After heating at 400 and 500 °C, the LEED patterns have germanene and superstructure spots, indicating that the crystal structure of germanene has been recovered (Figure 2a). The acceleration electron energy dependence of the LEED patterns of as‐grown and reformed germanene are shown in Figure S10 (Supporting Information). Since the O 1*s* peak disappeared completely upon heating to 350 °C (Figure 2b), no residual oxygen was incorporated in the reformed germanene. The GeO_x_ peaks also disappeared upon heating to 350 °C (Figure 2c), which agrees with the disappearance of the O 1*s* peak in Figure 2b. The Ge 3*d* spectra after heating at 400 and 500 °C show the same peak shape and chemical shift as the as‐grown germanene (see also Figures S11 and S12, Supporting Information). These results indicate that UHV heating of oxidized germanene results in germanene reformation and that its quality, such as crystallinity, degree of oxygen contamination, and chemical shift of Ge, is equivalent to that of as‐grown germanene on Ag(111)/Ge(111).

On the other hand, the LEED patterns and SRXPS spectra after heating at 300 °C correspond to the Ag_2_Ge surface alloy phases^[^ ^31^ ^]^ with and without the so‐called stripped phase.^[^ ^15c,d^ ^]^ The formation of Ag_2_Ge surface alloy phases on Ag(111) has been reported at temperatures similar to those of our study.^[^ ^15c,d^ ^]^ This suggests that after the removal of oxygen from oxidized germanene, Ge segregates on the Ag(111) surface as well as on the surface without oxidation. In summary, the changes in the sample can be represented schematically in Figure 2d.

**Figure 3:**
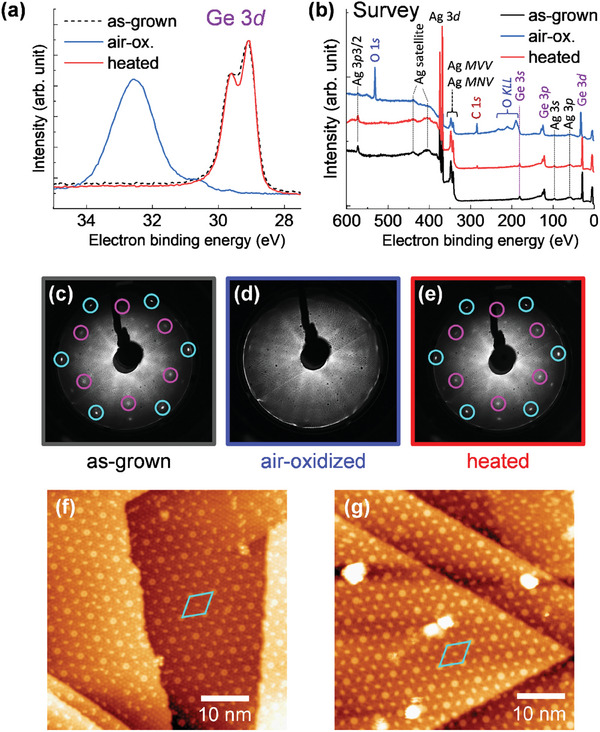
Air oxidation and reformation of germanene. a,b) SRXPS Ge 3*d* (a) and survey (b) spectra of germanene as‐grown (black), after air‐oxidized (blue), and after being air‐oxidized and heated (red). c–e) LEED patterns of germanene as‐grown (c), after air‐oxidized (d), and after being air‐oxidized and heated (e). The incident beam energy for LEED is 70 eV. f,g) Representative LTSTM images of as‐grown (f) and reformed germanene (g). Sample bias and tunneling current setpoint were 1.0 V and 500 pA for (f), and 1.0 V and 1.0 nA for (g), respectively. The blue rhomboidal shapes in (f) and (g) indicate the (7√7 × 7√7) R19.1° supercell with respect to Ag(111).

**Figure** **3** shows air oxidation and reformation of germanene. Figure 3a,b shows Ge 3*d* and survey SRXPS spectra of as‐grown, air‐oxidized, and reheated germanene, respectively. The air exposure time was >2 h, and the UHV heating temperature was 500 °C, which is the typical growth temperature of germanene. It is noted that no Ar^+^ ion sputtering was performed on the sample after the air exposure. Similar Ge 3*d* peaks were observed both before air exposure and after reheating (Figure 3a). The survey spectra also show similar peaks for the as‐grown and reheated germanene (Figure 3b). One difference between the two is the presence of C 1*s*. This suggests that air exposure leads to surface carbon contamination, which is partially eliminated by a standard UHV heating process at ≈500 °C. Figure 3c–e shows LEED patterns of as‐grown, air‐oxidized, and reheated germanene, respectively. The LEED patterns of air‐oxidized germanene were fully recovered after UHV heating, as was recovered from O_2_ oxidation (Figure 2a), indicating that the crystal structure of germanene was recovered.

Figure 3f,g is representative LTSTM images of as‐grown germanene and reheated germanene after air oxidation, respectively. The STM image of as‐grown germanene (Figure 3f) shows a (7√7 × 7√7) R19.1° supercell with respect to Ag(111), which corresponds to the previously reported surface structure of germanene on Ag(111)/Ge(111).^[^ ^15a^ ^]^ Since the same (7√7 × 7√7) R19.1° structure was observed over a large area of the reheated sample after air oxidation (Figure 3g), the reformed germanene has high uniformity and exactly the same atomic structure as the as‐grown germanene. The large and bright spots identified in the STM image (Figure 3f) are likely to be carbon contaminants, as was detected in the SRXPS spectrum (red spectrum in Figure 3b). From the above, it is clear that oxidized germanene, including air‐oxidized one, can be recovered to its original germanene structure by a simple UHV heating at ≈500 °C.

**Figure 4:**
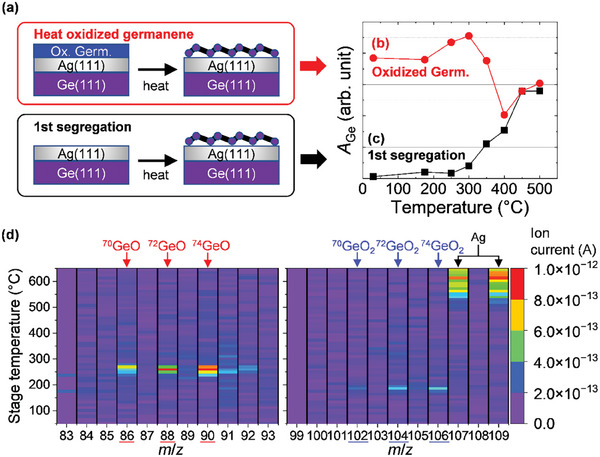
a–c) Heating temperature dependence of the peak areas of Ge 3*d* (*A*_Ge_) on oxidized germanene (b) and Ag(111)/Ge(111) surface (c), and its illustration (a). The Ge 3*d* spectra were obtained at RT. Note that the Ag(111)/Ge(111) surface was sputtered by Ar^+^ ions with a kinetic energy of 2 keV for 20 min to remove surface contaminants before the SRXPS measurements. d) Ion current mapping as a function of heating temperature for various *m*/*z* obtained from air‐oxidized germanene. The *m*/*z* ranges are 83–93 (left) and 99–109 (right). Among the naturally existing Ge isotopes (^70^Ge, ^72^Ge, ^73^Ge, ^74^Ge, ^76^Ge), GeO and GeO_2_ desorption of ^70^Ge, ^72^Ge, and ^74^Ge, which are relatively abundant in nature, were observed at ≈250 and ≈200 °C, respectively.

To elucidate the mechanism of the germanene reformation on Ag(111)/Ge(111) from oxidized germanene by O_2_ backfilling and air exposure, the heating temperature dependence of SRXPS spectra of the sample surfaces was investigated. **Figure** **4**b,c shows the dependence of Ge 3*d* peak areas (*A*_Ge_), corresponding to the amount of surface Ge, on heating temperatures for oxidized germanene and as‐deposited Ag(111)/Ge(111) surface, respectively. The *A*_Ge_ of Ag(111)/Ge(111) shows a monotonic increase when the heating temperature exceeds 300 °C, indicating that the surface segregation of Ge began at 300 °C (Figure 4c). On the other hand, the *A*_Ge_ of the oxidized germanene showed a non‐monotonic tendency with respect to the heating temperature. The *A*_Ge_ first increased slightly from 250 to 300 °C, then decreased from 300 to 400 °C, and finally increased again above 400 °C (Figure 4b). The increase in *A*_Ge_ of oxidized germanene from 250 to 300 °C and above 400 °C can be explained by Ge segregation since the 1st segregation starts at a similar temperature (≈300 °C) (Figure 4c). On the other hand, the trend of *A*_Ge_ decreasing from 300 to 400 °C in oxidized germanene is difficult to explain from the above results. A possible hypothesis for the decrease in the surface Ge is either desorption to the vacuum side or diffusion into the Ag side.

TDS measurements were performed to verify the reason for the decrease in the surface Ge of the oxidized germanene on heating from 300 to 400 °C. Air‐oxidized samples were used for the TDS measurements. Figure 4d shows ion current mapping of air‐oxidized germanene as a function of heating temperature for various mass‐to‐charge ratios (*m*/*z*). Three peaks were observed around 250 °C for *m*/*z* of 86, 88, and 90, and another three peaks around 200 °C for *m*/*z* of 102, 104, and 106. Considering the relatively abundant natural existing Ge isotopes (**Table** **1**), these peaks were assigned to GeO (^70^GeO, ^72^GeO, and ^74^GeO) and GeO_2_ (^70^GeO_2_, ^72^GeO_2_, and ^74^GeO_2_). The desorption of O_2_ was below the detection limit (discussed in detail in Figures S7 and S8, Supporting Information). Note that the stage temperatures in Figure 4d were obtained by thermocouples inside the sample holder, which have a certain offset from the actual sample surface temperature. Taking account that the previously reported Ag evaporation temperature in the Ag/Ge system is ≈700 °C^[^ ^15b^ ^]^ and that the Ag evaporation was detected at ≈600 °C as a peak temperature in the present TDS, the actual surface temperature would be ≈100 °C higher than the TDS stage temperature (the vertical axis of Figure 4d). Therefore, the desorption peak temperatures of GeO and GeO_2_ are estimated to be 350 and 300 °C, respectively, which are in good agreement with the results of SRXPS, where oxygen was removed (Figure 2b,c) and surface Ge decreased (Figure 4b). These results indicate that the oxygen removal and surface Ge decrease from 300 to 400 °C, owing to the desorption of GeO and GeO_2_, resulting in the high‐quality germanene reformation without residual oxygen as well as the ordinary Ge segregation from Ag(111)/Ge(111). It should be noted that the transformation of surface Ge on Ag(111)/Ge(111) by oxidation and annealing in this study is not due to thermal reduction but to the reformation of germanene by the desorption of oxidized Ge followed by Ge re‐segregation.

**Table 1:** Partial list of Ge isotopes. Only naturally abundant nuclides are listed.

Nuclide	Natural Abundance [%]
^70^Ge	20.5
^72^Ge	27.4
^73^Ge	7.7
^74^Ge	36.5
^76^Ge	7.7

**Figure 5:**
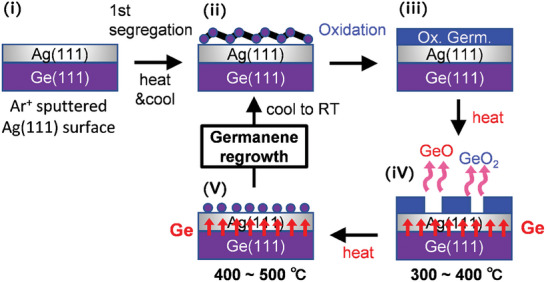
Intuitive schematic of the whole process of the oxidation and reformation of germanene on Ag(111)/Ge(111). Clean Ag(111) surface on Ge(111) is prepared by Ar^+^ ion sputtering (i). Segregated growth of germanene by heating and cooling in UHV (ii). Oxidation of germanene by exposure to O_2_ or air at RT (iii). Complete removal of oxygen by the desorption of GeO and GeO_2_ (iV). Heating‐induced Ge re‐segregation leads to germanene reformation (V).

**Figure** **5** shows an intuitive schematic of the whole process of the oxidation and reformation of germanene on Ag(111)/Ge(111). As mentioned above, the key reaction is the removal of oxygen by the evaporation of GeO and GeO_2_ (Figure 5iv). Once the oxygen is completely removed, Ge re‐segregation occurs as in the 1st segregation, followed by the regrowth of germanene (Figure 5V). Since the quality of the reformed germanene is equivalent to that of the initially segregated germanene, the germanene sample can be used repeatedly without Ar^+^ sputtering. This is primarily beneficial for basic research on germanene, especially in the field of surface science. Practically, the GeO and GeO_2_ desorption can play a role in increasing the purity of germanene. Although it is necessary to simultaneously compensate for the lost Ge, such a high purification of germanene will be beneficial for improving or mediating germanene device properties in the future.

**Figure 6:**
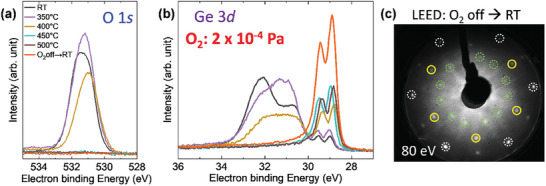
O_2_ exposure to germanene at high temperatures. a,b) SRXPS spectra of O 1*s* (a) and Ge 3*d* (b) were acquired at elevated temperatures with a *P*_O2_ of 2 × 10^−4^ Pa. The red spectra in (a,b) were recorded at RT after stopping the O_2_ exposure at 500 °C followed by cooling of the sample. c) LEED pattern at RT for the sample corresponding to the red spectra in (a,b). The clear spots indicate the reformation of germanene.

Next, we subjected germanene to oxygen exposure at high temperatures. The motivation is high‐temperature oxidation to form more robust germanium oxide (GeO_2_) on germanene. The technique of forming a clean GeO_2_/germanene interface should be beneficial for fabricating germanene‐based field effect transistors. **Figure** **6**a,b shows SRXPS spectra of O 1*s* and Ge 3*d* obtained at different temperatures under the O_2_ pressure (*P*_O2_) of 2 × 10^−4^ Pa, respectively. The O 1*s* peak was observed at 400 °C but disappeared above 450 °C (Figure 6a). The oxidation state of Ge 3*d* varied with temperature and disappeared above 450 °C (Figure 6b), which is in good accordance with O 1*s*. When the O_2_ backfilling was stopped at 500 °C, and the sample was then cooled to RT, the typical Ge 3*d* of germanene was observed (red spectrum in Figure 6b). The obtained LEED pattern of the sample also shows a typical pattern of germanene on Ag(111)/Ge(111) (Figure 6c), indicating that germanene reformation occurred even under a low‐pressure O_2_ atmosphere (2 × 10^−4^ Pa).

It has been well known that GeO desorption occurs in GeO_2_ thin films on Ge bulk crystals at temperatures above 400 °C.^[^ ^32^ ^]^ In this system, the presence of the GeO_2_/Ge interface is an important trigger, as the oxygen vacancies formed at the interface diffuse to the top surface, resulting in GeO desorption.^[^ ^32^, ^33^ ^]^ It is also known that this GeO desorption can be effectively suppressed by high‐pressure O_2_ oxidation (HPO), roughly 200 atm at 600 °C, forming an excellent GeO_2_/Ge gate stack.^[^ ^32^, ^34^ ^]^ This suggests that a stable, atomically thin Ge oxide or Ge oxide/germanene interface on Ag(111)/Ge(111) could be achieved when the O_2_ pressure is considerably higher.

Compared to the conventional GeO_2_/Ge system, the present GeO_x_ desorption from oxidized germanene on the Ag(111)/Ge(111) shows two noticeable phenomena. One is the low‐temperature desorption of GeO, and the other is the occurrence of GeO_2_ desorption. The desorption of GeO in the GeO_2_/Ge system usually occurs above 500 °C, which is recorded as the peak temperature of TDS.^[^ ^32a–c^ ^]^ The lowest reported GeO desorption peak temperature is ≈450 °C for ozonated Ge oxides,^[^ ^32a^ ^]^ indicating that the GeO desorption in our study is ≈100 °C lower. This low‐temperature desorption of GeO probably originates from the atomically thin nature of the Ge oxide. The previous studies revealed that the GeO desorption temperature increases with the increasing thickness of the GeO_2_ layer on Ge, which is due to the increase in the diffusion length of the oxygen vacancy required for the GeO desorption.^[^ ^32^, ^33^ ^]^ In the case of the present oxidized germanene, the atomically thin Ge oxides do not require the diffusion of oxygen vacancies for GeO desorption, thereby resulting in substantially low‐temperature desorption of GeO (≈350 °C).

Another plausible explanation for the low‐temperature desorption of GeO is the catalytic effect of the Ag(111) surface. Oniki et al. reported that the peak desorption temperatures of GeO from Al/GeO_2_ and Au/GeO_2_ thin film stacks on SiO_2_ decreased to ≈400 and ≈430 °C, respectively, due to the catalytic effect of metals.^[^ ^32d^ ^]^ Although we could not find any reports on the Ag‐induced lowering of GeO desorption temperature, since Ag, Al, and Au are commonly used metals for metal‐induced crystallization of amorphous Ge,^[^ ^35^ ^]^ we expect that similar catalytic effects of Ag on GeO desorption occurred. As mentioned above, the atomic thinness of oxidized germanene is attributed to the lower desorption temperature of GeO, and hence, the catalytic effect of Ag would have further lowered the desorption temperature of GeO.

On the other hand, GeO_2_ desorption was rarely reported.^[^ ^32^, ^36^ ^]^ This may be because the primary research interest is focused on forming excellent GeO_2_/Ge gate stacks, leading to the studies of GeO desorption rather than GeO_2_. There are two possible reasons for the occurrence of GeO_2_ desorption from oxidized germanene. One is the absence of the GeO_2_/Ge interface in the present case. When a GeO_2_/Ge interface exists, GeO_x_ with Ge^3+^ and Ge^4+^ is reduced to Ge^2+^ state, followed by GeO desorption. The absence of GeO_2_/Ge interfaces prevents the reduction of GeO_2_ to GeO, resulting in GeO_2_ desorption. The other reason is the higher vapor pressure of GeO_2_ than GeO below ≈740 K (≈467 °C).^[^ ^37^ ^]^ Zhang and Xu reported that the vapor pressures of GeO_2_ and GeO reach 1 × 10^−7^ Pa at ≈655 K (≈382 °C) and ≈700 K (≈427 °C), respectively.^[^ ^37^ ^]^ This higher vapor pressure of GeO_2_ at low temperatures reasonably explains why GeO_2_ is desorbed at lower temperatures than GeO.

As mentioned above, HPO is one promising way of producing GeO_2_ on germanene, but it is generally impossible to introduce high‐pressure O_2_ (above 10 atm) into a UHV chamber while maintaining sample preparation and analysis capabilities. Instead of the HPO, we focused on the supersonic molecular beam (SSMB) as an alternative method, which can accelerate gas‐surface reactions with high activation barriers.^[^ ^38^ ^]^ Here, germanene was exposed to O_2_ SSMB^[^ ^39^ ^]^ at high temperatures to extremely promote oxidation, as shown in Figure S5 (Supporting Information). However, the results indicate that even O_2_ SSMB did not suppress the GeO_x_ desorption. When the sample was cooled to RT after turning off the SSMB, the same LEED pattern as in Figure 6 was obtained, indicating that germanene was formed. Although the further control of Ge oxide formation on germanene requires more effort, the instability of oxidized germanene/Ag(111)/Ge(111) at high temperatures provides the robustness for the present germanene reformation process as well as the germanene segregation process on Ag(111)/Ge(111).

## 3 Conclusion

In conclusion, we have found the heating‐induced reformation of germanene on Ag(111)/Ge(111) from its oxidized phase and have clarified the mechanism. The revealed key reaction was the removal of oxygen by the desorption of GeO and GeO_2_ at ≈300—400 °C, leading to the germanene reformation by the further segregation of Ge. The reformed germanene has a (7√7 × 7√7) R19.1° supercell with respect to Ag(111) and does not contain residual oxygen, indicating that the quality of the reformed germanene is equivalent to the initially segregated one. Oxidized and reformed germanene can be used repeatedly without Ar^+^ ion sputtering, which is beneficial for basic research on germanene, especially in the field of surface science. Even after air oxidation, germanene can be easily reformed with simple UHV annealing. Our investigation also revealed that forming a clean GeO_2_/germanene interface is difficult due to the instability of oxidized germanene at high temperatures. However, this instability of oxidized germanene also indicates that the growth of germanene on Ag(111)/Ge(111) has a high tolerance to oxygen impurities, leading to a robust growth process. This work should provide a fundamental understanding of annealing effects not only on oxidized germanene but also on related group 14 2D materials, particularly segregated systems, such as germanene on Ag(111)/Ge(111) and Al(111)/Ge(111).

## 4 Experimental Section

### The Preparation of Ag(111)/Ge(111)

Commercially available undoped Ge(111) wafers were used as starting material. The Ge(111) wafers were cut into 30 × 3.4—10 mm^2^ chips to be placed on a sample holder for the MBE apparatus at the Japan Atomic Energy Agency (JAEA). The Ge(111) samples were introduced into the MBE chamber, followed by degassing, Ar^+^ ion sputtering, and annealing. The base pressure of the MBE chamber is <2.4 × 10^−7^ Pa. The Ar^+^ ion sputtering was performed using a SPECS IQE 11/35 at the Ar pressure of ≈1.33 × 10^−4^ Pa and an emission current of 10 mA. The detailed Ar^+^ ion sputtering and annealing process for Ge(111) samples consists of steps: sputtering at RT (1), sputtering during temperature rise from RT to ≈700 °C (2), sputtering at ≈700 °C (3), and annealing at ≈700 °C (4). In step (1), Ar^+^ ion sputtering at a kinetic energy of 2 keV was performed for 10 min at RT. In step (2), the sample temperature was increased by applying DC current while simultaneously sputtering by Ar^+^ ions at a kinetic energy of 2 keV. In step (3), the process sequence of Ar^+^ ion sputtering at different acceleration voltages was performed at ≈700 °C. The typical sequence of the sputtering was three sets of 2 and 1 keV sputtering for 45 and 5 min, respectively, followed by one set of 2, 1.5, and 1 keV sputtering for 30 min each. In step (4), the sample was annealed for 10 min at ≈700 °C. The resulting Ge(111) surface was observed by ex situ atomic force microscopy in air, as shown in Figure S6 (Supporting Information). The step and terrace structure was clearly observed, indicating that a clean and smooth surface of Ge(111) was developed.

Ag was subsequently deposited on the cleaned Ge(111) surface using an electron beam evaporator in the same MBE chamber. The deposition rate and thickness of Ag were typically ≈1.0 Å s^−1^ and ≈150 nm, respectively.

### Typical Growth Condition of Germanene on Ag(111)/Ge(111)

The prepared Ag(111)/Ge(111) samples were taken out from the MBE chamber and then transported in air from JAEA to SPring‐8 for SRXPS measurements. Typically, the samples were exposed to air for several days to several weeks. After introducing the samples into the UHV chamber (the base pressures < 10^−7^ Pa) for SRXPS measurements, the Ag surface was sputtered by Ar^+^ ions at 2 keV for 20 min to remove surface contamination. Subsequently, germanene was grown by annealing at ≈500 °C for 30 min in the UHV chamber. The growth of germanene was confirmed by LEED, as shown in Figure 1e.

### O_2_ Backfilling

The O_2_ backfilling was performed using a variable leak valve.

### SRXPS and Binding Energy Calibration for the Spectral Analysis

For the SRXPS measurements, a surface reaction analysis apparatus (SUREAC2000) built at BL23SU in SPring‐8 was used. The photoelectron was obtained at a normal angle to the surface. The photon energy of the monochromatic SR beam was 700 eV. The beam energy was calibrated by the Fermi energy of an Au sample.

### LEED

ErLEED (SPECS) was used to obtain LEED patterns on the sample surface.

### LTSTM

LTSTM observations were performed with an LTSTM (Omicron GmbH) maintained at ≈7 K under UHV (≈1.0 × 10^−8^ Pa). Electrochemically etched W tips were used for the STM imaging of germanene.

### TDS

TDS measurements were performed using TDS WA1000K (ESCO, Ltd.). The heating rate was 60 K min^−1^, and the base pressure of the TDS chamber was ≈4 × 10^−8^ Pa.

## Conflict of Interest

The authors declare no conflict of interest.

## Supporting information



Supporting Information

## Data Availability

The data that support the findings of this study are available from the corresponding author upon reasonable request.

## References

[1] a) P. Vogt , P. De Padova , C. Quaresima , J. Avila , E. Frantzeskakis , M. C. Asensio , A. Resta , B. Ealet , G. Le Lay , Phys. Rev. Lett. 2012, 108, 155501; 22587265 10.1103/PhysRevLett.108.155501

[2] M. Dávila , L. Xian , S. Cahangirov , A. Rubio , G. Le Lay , New J. Phys. 2014, 16, 095002.

[3] a) F.‐f. Zhu , W.‐j. Chen , Y. Xu , C.‐l. Gao , D.‐d. Guan , C.‐h. Liu , D. Qian , S.‐C. Zhang , J.‐f. Jia , Nat. Mater. 2015, 14, 1020; 26237127 10.1038/nmat4384

[4] a) J. Yuhara , B. He , N. Matsunami , M. Nakatake , G. Le Lay , Adv. Mater. 2019, 31, 1901017; 10.1002/adma.201901017 31074927

[5] A. Molle , J. Goldberger , M. Houssa , Y. Xu , S.‐C. Zhang , D. Akinwande , Nat. Mater. 2017, 16, 163. 28092688 10.1038/nmat4802

[6] Z. Ni , Q. Liu , K. Tang , J. Zheng , J. Zhou , R. Qin , Z. Gao , D. Yu , J. Lu , Nano Lett. 2011, 12, 113. 22050667 10.1021/nl203065e

[7] X.‐S. Ye , Z.‐G. Shao , H. Zhao , L. Yang , C.‐L. Wang , RSC Adv. 2014, 4, 21216.

[8] a) M. Ezawa , Phys. Rev. B. 2013, 87, 155415;

[9] B. Weber , M. S. Fuhrer , X.‐L. Sheng , S. A. Yang , R. Thomale , S. Shamim , L. W. Molenkamp , D. Cobden , D. Pesin , H. J. W. Zandvliet , P. Bampoulis , R. Claessen , F. R. Menges , J. Gooth , C. Felser , C. Shekhar , A. Tadich , M. Zhao , M. T. Edmonds , J. Jia , M. Bieniek , J. I. Väyrynen , D. Culcer , B. Muralidharan , M. Nadeem , J. Phys. Mater. 2024, 7, 022501.

[10] A. Zhao , B. Wang , APL Mater. 2020, 8, 030701.

[11] a) C.‐C. Liu , W. Feng , Y. Yao , Phys. Rev. Lett. 2011, 107, 076802; 21902414 10.1103/PhysRevLett.107.076802

[12] A. Acun , L. Zhang , P. Bampoulis , M. Farmanbar , A. van Houselt , A. Rudenko , M. Lingenfelder , G. Brocks , B. Poelsema , M. Katsnelson , J. Phys.: Condens. Matter. 2015, 27, 443002. 26466359 10.1088/0953-8984/27/44/443002

[13] a) Y. Xu , B. Yan , H.‐J. Zhang , J. Wang , G. Xu , P. Tang , W. Duan , S.‐C. Zhang , Phys. Rev. Lett. 2013, 111, 136804; 24116803 10.1103/PhysRevLett.111.136804

[14] S. Konschuh , M. Gmitra , J. Fabian , Phys. Rev. B. 2010, 82, 245412.

[15] a) J. Yuhara , H. Shimazu , K. Ito , A. Ohta , M. Araidai , M. Kurosawa , M. Nakatake , G. Le Lay , ACS Nano. 2018, 12, 11632; 30371060 10.1021/acsnano.8b07006

[16] M. E. Dávila , G. Le Lay , Sci. Rep. 2016, 6, 20714. 26860590 10.1038/srep20714 PMC4748270

[17] Z. Qin , J. Pan , S. Lu , Y. Shao , Y. Wang , S. Du , H. J. Gao , G. Cao , Adv. Mater. 2017, 29, 1606046. 10.1002/adma.201606046 28134451

[18] a) M. Derivaz , D. Dentel , R. Stephan , M.‐C. Hanf , A. Mehdaoui , P. Sonnet , C. Pirri , Nano Lett. 2015, 15, 2510; 25802988 10.1021/acs.nanolett.5b00085

[19] L. Li , S. z. Lu , J. Pan , Z. Qin , Y. q. Wang , Y. Wang , G. y. Cao , S. Du , H. J. Gao , Adv. Mater. 2014, 26, 4820. 24841358 10.1002/adma.201400909

[20] J. Yuhara , D. Matsuba , M. Ono , A. Ohta , S. Miyazaki , M. Araidai , S.‐i. Takakura , M. Nakatake , G. Le Lay , Surf. Sci. 2023, 738, 122382.

[21] L. Zhang , P. Bampoulis , A. Rudenko , Q. v. Yao , A. Van Houselt , B. Poelsema , M. Katsnelson , H. Zandvliet , Phys. Rev. Lett. 2016, 116, 256804. 27391741 10.1103/PhysRevLett.116.256804

[22] L. Persichetti , F. Jardali , H. Vach , A. Sgarlata , I. Berbezier , M. De Crescenzi , A. Balzarotti , J. Phys. Chem. 2016, 7, 3246. 10.1021/acs.jpclett.6b01284 27487453

[23] L. Tao , E. Cinquanta , D. Chiappe , C. Grazianetti , M. Fanciulli , M. Dubey , A. Molle , D. Akinwande , Nat. Nanotechnol. 2015, 10, 227. 25643256 10.1038/nnano.2014.325

[24] a) B. Madhushankar , A. Kaverzin , T. Giousis , G. Potsi , D. Gournis , P. Rudolf , G. Blake , C. Van Der Wal , B. Van Wees , 2D Mater. 2017, 4, 021009;

[25] a) S. Suzuki , K. K. H. De Silva , M. Yoshimura , T. Nakayama , Jpn. J. Appl. Phys. 2020, 59, SN1004;

[26] R. C. Longo , R. Addou , K. Santosh , J.‐Y. Noh , C. M. Smyth , D. Barrera , C. Zhang , J. W. Hsu , R. M. Wallace , K. Cho , 2D Mater. 2017, 4, 025050.

[27] a) Y. Zhu , S. Murali , W. Cai , X. Li , J. W. Suk , J. R. Potts , R. S. Ruoff , Adv. Mater. 2010, 22, 3906; 20706983 10.1002/adma.201001068

[28] M. Cavicchioli , L. C. Varanda , A. C. Massabni , P. Melnikov , Mater. Lett. 2005, 59, 3585.

[29] I. Ishigami , E. Tsunasawa , K. Yamanaka , Trans. Jpn. Inst. Met. 1981, 22, 337.

[30] M. Rogala , G. Bihlmayer , P. Dabrowski , C. Rodenbücher , D. Wrana , F. Krok , Z. Klusek , K. Szot , Sci. Rep. 2019, 9, 12563. 31467321 10.1038/s41598-019-48837-3 PMC6715630

[31] a) H. Oughaddou , S. Sawaya , J. Goniakowski , B. Aufray , G. Le Lay , J. Gay , G. Tréglia , J. Bibérian , N. Barrett , C. Guillot , A. Mayne , G. Dujardin , Phys. Rev. B. 2000, 62, 16653;

[32] a) A. Toriumi , T. Nishimura , Jpn. J. Appl. Phys. 2017, 57, 010101;

[33] S. K. Wang , K. Kita , T. Nishimura , K. Nagashio , A. Toriumi , Jpn. J. Appl. Phys. 2011, 50, 04DA01.

[34] a) C. H. Lee , T. Nishimura , K. Nagashio , K. Kita , A. Toriumi , IEEE Trans. Electron Devices. 2011, 58, 1295;

[35] G. Maity , S. Dubey , T. Meher , S. Dhar , D. Kanjilal , T. Som , S. P. Patel , RSC Adv. 2022, 12, 33899. 36505692 10.1039/d2ra06096e PMC9703449

[36] M. Kobayashi , G. Thareja , M. Ishibashi , Y. Sun , P. Griffin , J. McVittie , P. Pianetta , K. Saraswat , Y. Nishi , J. Appl. Phys. 2009, 106, 104117.

[37] L. Zhang , Z. Xu , J. Hazard. Mater. 2016, 312, 28. 27015376 10.1016/j.jhazmat.2016.03.025

[38] A. Al Taleb , F. Schiller , D. V. Vyalikh , J. M. Pérez , S. V. Auras , D. Farías , J. E. Ortega , Phys. Chem. Chem. Phys. 2024, 26, 1770. 38168970 10.1039/d3cp05071h

[39] a) Y. Tsuda , A. Yoshigoe , S. Ogawa , T. Sakamoto , Y. Takakuwa , e‐J. Surf. Sci. Nanotechnol. 2022, 21, 30;

