# Supplementary material for: Germanene Reformation from Oxidized Germanene on Ag(111)/Ge(111) by Vacuum Annealing
Source: Small Methods. 2024 Sep 9;9(3):2400863. doi: 10.1002/smtd.202400863 (PMC11926502; doi:10.1002/smtd.202400863)
Supplement: Supplementary file 1 — Supporting Information [file SMTD-9-2400863-s001.docx]

Supporting Information

Germanene reformation from oxidized germanene on Ag(111)/Ge(111) by vacuum annealing

Seiya Suzuki*, Daiki Katsube, Masahiro Yano, Yasutaka Tsuda, Tomo-o Terasawa, Takahiro Ozawa, Katsuyuki Fukutani, Yousoo Kim, Hidehito Asaoka, Junji Yuhara, and Akitaka Yoshigoe

1. Effects of synchrotron radiation (SR) and Bayard-Alpert (BA) hot cathode ionization gauges on germanene oxidation

We found that the oxidation of germanene on Ag(111)/Ge(111) is accelerated by the SR for SR X-ray photoelectron spectroscopy (SRXPS) and the two BA hot cathode ionization gauges to monitor chamber pressure. Figure S1 compares the effect of SR on the oxidation of germanene. The oxidation was performed at room temperature (RT) under the O_2_ pressure (*P*_O2_) of 2 × 10^-4^ Pa. Note that the oxidation to obtain the SRXPS spectra in Figure S1 was performed with the BA gauges in use.

Relatively similar Ge 3*d* spectra are intentionally shown with the same color (black, red, purple, green, and blue) in Figure S1a,c. Among the Ge 3*d* spectra, the spectra for 25 and 50 min with SR (Figure S1a) and for 100 and 160 min without SR (Figure S1c) are particularly similar, respectively, while the shapes of the corresponding spectra with and without SR are almost the same with and without SR. Therefore, we estimated the effect of SR on the oxidation acceleration to be 3~4 times.

Figure S2 compares the effect of BA gauges on the oxidation of germanene. Figure S2a,b shows Ge 3*d* and O 1*s* spectra of germanene oxidized under the conditions that minimize the effect of the BA gauges on oxidation. In these cases, *P*_O2_ was monitored by a BA gauge only from the start of backfilling until reaching the target *P*_O2_ (roughly 30 sec to 1 min), and then the BA gauge was turned off during the oxidation. The *P*_O2_ after keeping the oxidation time was checked several times, and the difference from the initial target *P*_O2_ was within about 10%, indicating that it is stable enough to estimate the effects of the BA gauges for the accelerating germanene oxidation. Hereafter, we call the aforementioned oxidation “oxidation without (w/o) BA,” while normal oxidation in which the BA gauges are kept on during the O_2_ exposure is referred to as “oxidation with BA.” Figure S2c,d shows the Ge 3*d* and O 1*s* spectra of the germanene oxidized with BA. The Ge 3*d* and O 1*s* spectra in Figure S2c,d are the same as in Figure S1c,d, respectively, but the accumulated exposure times have been converted to O_2_ dose in Langmuir (L) unit to facilitate comparison with Figure S2a,b. For the conversion of O_2_ exposure in the L unit, the equation that 1 L is equal to 1.33 × 10^-4^ Pa∙s was used.

Focusing on the transition of Ge 3*d* w/o BA due to O_2_ dosing (Figure S2a), the oxidation of germanene saturates between 440 ~ 650 kL. Since the oxidation with BA gauges saturated ~ 25 kL, it is clear that the oxidation of germanene was accelerated by the BA gauges. The BA gauges also affect the oxidation state of germanene. While the new peak around 32 eV in Ge 3*d* at large O_2_ doses arose from the oxidation with BA (Figure S2c), only a tail-like feature around 32 eV can be seen in the case of the oxidation w/o BA (Figure S2a). The difference in oxidation state with and w/o BA can also be seen in O 1*s*, where the O 1*s* w/o BA shifts only slightly to the high binding energy with increasing O_2_ dose, but that of the O 1*s* with BA shows a large shift. The oxidation state of germanene accelerated by the BA gauges is similar to that of germanene oxidized in air (as discussed in Figure 3 and Figure S3), indicating that the oxidation state of this germanene is close to the naturally stable one.

The requirement of a considerably large dose of O_2_, ~ 500 kL (Figure S2a,b), to complete the oxidation of germanene indicates that the intrinsic oxidation of germanene by an O_2_ molecule occurs at an extremely low probability at RT.

1. Discussion on oxidation state of germanene

To discuss the difference in oxidation state, Ge 3*d* spectra of 3 types of germanene, being fully oxidized by O_2_ with SR, without SR, and by air, were compared as shown in Figure S3a. The Ge 3*d* spectrum of the air-oxidized germanene is apparently shifted to the high binding energy, while the Ge 3*d* spectrum of germanene oxidized by O_2_ with and without SR are both similar. To perform the peak fitting of the Ge 3*d* spectra of oxidized germanene, we first subjected the Ge 3*d* spectrum of as-grown germanene on Ag(111)/Ge(111). Figure S4 shows the Ge 3*d* spectrum of as-grown germanene and its fitted curves for Ge 3*d*_5/2_, 3*d*_3/2_, and their sum. The Ge 3*d* spectrum of as-grown germanene was first subtracted by a Shirley background. Since the Ge 3*d* spectrum of germanene has two peaks of Ge 3*d*_5/2_ and 3*d*_3/2_, two peaks were fitted separately with a pseudo-Voigt function. The obtained fitting parameters of the Ge 3*d* of as-grown germanene on Ag(111)/Ge(111) are shown in Table S1.

To fit the Ge 3*d* spectra of oxidized germanene, a sum of the Ge 3*d*_5/2_ and 3*d*_3/2_ curves with the fixed separation energy and peak area ratio of 0.57 eV and 0.73 between Ge 3*d*_5/2_ and 3*d*_3/2_, respectively, was used as the standard peak. To simplify the fitting and to focus on the chemical shift at particularly high binding energies in Ge 3*d*, the fitting region was limited to higher than 29.8 eV. The multiple standard peaks between 2 to 4 as fitting curves for the Ge 3*d* of oxidized germanene were considered and tested, and we found that 2 standard peaks are sufficiently reasonable to discuss the difference between Ge 3*d* of O_2_-oxidized and air-oxidized germanene.

Figure S3b,c shows the results of the Ge 3*d* fitting for the O_2_-oxidized and air-oxidized germanene. Ge 3*d*_5/2_ peaks were observed at 30.47 and 31.87 eV for O_2_-oxidized germanene and 32.33 and 30.29 eV for air-oxidized germanene. To assign the oxidation numbers of the Ge in the oxidized germanene, we obtained the relative core level shifts of Ge 3*d*_5/2_ with respect to that of as-grown germanene. The obtained relative core level shifts are listed with the corresponding plausible oxidation numbers of Ge in Table S2. Compared to the core level shifts of the oxidized bulk Ge(111) surface,^[1]^ the individual shifts are slightly different, but the average shift per oxygen atom is close (0.83 and 0.85 eV). Since the core level shift of the Ge 3*d*_5/2_ for air-oxidized germanene shows a higher energy shift than for dry-O_2_ oxidized germanene and supposedly corresponds to Ge^4+^, H_2_O molecules may influence the oxidation state of germanene. Or, considering the Boltzmann distribution, the hardly existing high energy O_2_ at RT may be related to the oxidation of germanene to the Ge^4+^ state. Although the present results provide important information about the oxidation state of germanene, further experiments and analyses will be necessary to discuss the oxidation state of germanene on Ag(111)/Ge(111) in greater depth and detail.

1. O_2_ supersonic molecular beam (SSMB) irradiation of germanene at high temperatures

*In situ* SRXPS measurement of O_2_ SSMB irradiation of germanene on Ag(111)/Ge(111) at high temperatures was performed to investigate the stability of oxidized germanene at high temperatures. The O_2_ SSMB was generated continuously by the adiabatic expansion of a mixture of O_2_ and He. The nozzle temperature at the aperture was kept at 1400 K to produce an SSMB with a translational energy of ~2.2 eV. The flux of the SSMB was evaluated by using a quadrupole mass spectrometer by separate measurements of the O_2_ and He fluxes. The flow rates of O_2_ and He were set at 2 and 198 sccm, respectively. The obtained O_2_ and He fluxes were 1.68 × 10^15^ and 5.13 × 10^15^ cm^-2^∙s^-1^, respectively.

Figure S5 shows the results of the O_2_ SSMB irradiation of germanene at high temperatures. Figure S5a shows the temperature transition (left) and color-scaled photoelectron intensity around Ge 3*d* (right) over irradiation time. Note that the color scale for the intensities is normalized (red for the highest and blue for the lowest) for each irradiation time in Figure S5a. Figure S5b shows Ge 3*d* SRXPS spectra at different irradiation times, also corresponding to different temperatures. Before O_2_ SSMB irradiation, the temperature was kept at 350 °C (Figure S5b-1). Immediately after the start of the O_2_ SSMB irradiation, the Ge 3*d* shifted to 31 to 33 eV (can be seen as red color in Figure S5), indicating the oxidation of germanene at 350 °C (Figure S5b-2). By increasing the temperature to 400 °C with O_2_ SSMB irradiation, the oxidation state of germanene was changed to Figure S5-3. The decrease in intensity around 31 eV at 400 °C would be due to the desorption of GeO or disproportionation of GeO to Ge and GeO_2_.^[2]^ Further increase in the heating temperature leads to a decrease in the oxidized state of Ge 3*d*. The oxidized state disappeared at 500 °C even under the O_2_ SSMB irradiation (Figure S5-6), and finally, germanene can form after stopping the O_2_ SSMB irradiation followed by cooling to RT.

1. *Ex situ* atomic force microscopy (AFM) observation of sputtered and annealed Ge(111) surface

Surface morphology of cleaned Ge(111) was observed in air using AFM (Shimazu, SPM-8100FM) in dynamic force mode. Commercially available Si cantilevers with a spring constant of ~42 N/m and a resonant frequency of ~ 320 kHz were used.

Figure S6a shows the typical AFM image of the cleaned Ge (111) surface. The step-and-terrace structure was clearly observed. Figure S6b shows the line profiles obtained in Figure S6a. Step heights below 0.6 nm were observed. Since the one atomic step of Ge(111) is ~0.327 nm, the smallest steps observed in Figure S6 are one atomic layer.

1. Investigating the possibility of direct desorption of O_2_ from oxidized germanene during heating

Additional TDS analyses and an *in situ* TDS experiment were performed to prove the desorption of GeO and GeO_2_ further and investigate the possibility of direct desorption of O_2_ from oxidized germanene during heating.

Figure S7 shows the added TDS analyses of air-oxidized germanene. Figure S7a compares *m/z* 32, 90, and 106 TDS spectra. The *m/z* 90 and 106 correspond to ^74^GeO and ^74^GeO_2_, as is already discussed in Figure 4. The GeO desorption occurred around 290 °C. Still, no peak occurred around the same temperature for *m/z* 32, indicating that there is no desorption of O_2_ during GeO desorption. On the other hand, the TDS peak of *m/z* 32 and ^74^GeO_2_ (*m/z* 106) are found around 220 °C. Still, these peak temperatures are slightly different (220 and 215 °C for *m/z* 32 and ^74^GeO_2_, respectively), indicating that these peaks originated from different desorption events.

To obtain more information about the TDS peak at *m/z* 32, including whether or not it is due to O_2_ desorption, we compared the TDS spectra at *m/z* 32-35 as shown in Figure S7b. It was found that the TDS peak at *m/z* 32-34 has quite a similar peak but is different from *m/z* 35, indicating that the TDS peak at *m/z* 32-34 around 220 °C is the same origin. The most plausible origin for *m/z* 32-34 is the desorption of H_2_S. The *m/z* 34 signal is from H_2_S, and the *m/z* 33 and 32 are HS and S fragment ions derived from H_2_S.

The origin of the sulfur in the desorbed H_2_S is probably the sulfurization of the Ag surface in the air.^[3]^ Our sample for TDS measurements was stored in air for several weeks after preparation; then, the TDS measurements were performed in UHV. Therefore, sulfurization of the Ag surface in the air is difficult to avoid in such *ex situ* TDS measurements.

To overcome the disadvantage of *ex situ* TDS, we performed *in situ* TDS measurement for oxidized germanene on Ag(111)/Ge(111). Germanene was grown in a UHV chamber, and subsequent oxidation and TDS were performed in the same chamber. The oxidation of germanene was conducted at 150 °C by exposure 300 kL of O_2_ gas. Figure S8 shows the result of *in situ* TDS. Although GeO_2_ desorption was not observed, probably due to the sensitivity of the experimental setup, GeO desorption was observed around 380 °C at *m/z* 90. Since the TDS spectrum at *m/z* 32 shows no peak during the TDS, the desorption of O_2_ from oxidized germanene is below the detection limit.

1. Discussion on the Ge 3*d* spectra change of oxidized germanene at low temperatures

As shown in Figure 2b, oxygen disappeared after heating at 350 °C thanks to the desorption of GeO and GeO_2_. Since germanene growth occurs at 500 °C, the surface becomes oxygen-free above 350 °C, regardless of whether the starting surface is Ag(111) or oxidized germanene/Ag(111) on Ge(111). As a result, the reformed germanene from its oxidized phase is of equivalent quality to the as-grown germanene, as can be seen from the LEED (Figure 2a and 3e) and XPS (Figure 2b,c and 3a). Thus, the desorption of GeO and GeO_2_ contributes to the removal of oxygen but does not contribute to the resulting structure of reformed germanene.

On the other hand, oxidized germanene still contains oxygen after heating at 250 and 300 °C (Figure 2b). This oxidized germanene contains mainly Ge^3+^ and Ge^2+^ oxidation states (Figure 2c and S9). As shown in Figure S9, the Ge^3+^ state decreases at low temperatures (<250 °C), followed by a significant decrease in the Ge^2+^ state (~300 °C). At even higher temperatures (>350 °C), the Ge^3+^ and Ge^2+^ states decrease and disappear. Since the oxidation number of Ge in GeO is Ge^2+^, the decrease of Ge^2+^ in the Ge 3*d* spectrum at higher temperatures (~300 °C) directly corresponds to the GeO desorption event. Meanwhile, the decrease of Ge^3+^ in the Ge 3*d* spectra at low temperatures (~250 °C) is probably related to the desorption of GeO_2_. However, the reaction event from the Ge^3+^ state in oxidized germanene to the desorption of GeO_2_ would be complicated because their oxidation numbers are different. The increase in Ge^2+^ state at 250 °C may indicate the reduction of Ge^3+^ or the disproportionation reaction from Ge^3+^ state to Ge^2+^ and GeO_2_.

1. Detail comparison of as-grown and reformed germanene by LEED and SRXPS

LEED patterns for different acceleration electron energies of as-grown and reformed germanene are shown in Figure S10. The unique diffraction spots shown in Figure S10 appear to be consistent with the first report of segregated germanene on Ag(111)/Ge(111) by Yuhara *et al*.^[4]^ In particular, the LEED patterns of 70, 30, and 20 eV in Figure S10 are better suited to judge the agreement with the reference.^[4]^ Thus, the LEED patterns indicate the formation of germanene with the surface supercell structure of (7√7 × 7√7)R19.1°.

The survey and Ge 3*d* SRXPS spectra of as-grown and reformed germanene are shown in Figure S11.

Figure S12 shows the Ge 3*d* SRXPS spectra of germanene/Ag(111)/Ge(111) with the photoelectron detecting angle of 70 degree from normal to the surface. The larger angle from the normal to the surface provides more surface-sensitive information in the photoelectron, thus allowing the chemical states of the topmost surface to be determined with greater precision. As shown in Figure S12, the peak top of the Ge 3*d*_5/2_ was located at 29.1 eV, which is smaller than that of Ge(111) clean surface (29.4 eV), and more extensive than that of Ag_2_Ge surface alloy (28.6 eV). It is close proximity to the reported segregated germanene (29.01 eV).^[4]^ Since chemical shifts reflect chemical bonding states well, surface-sensitive Ge 3*d* SRXPS shows the formation of germanene.

1. *Ex situ* Raman spectroscopy with hexagonal boron nitride (hBN)-capped segregated germanene

Figure S13a shows Raman spectra of hBN-capped germanene on Ag(111)/Ge(111) and bulk Ge. In-plane and out-of-plane vibrational modes of germanene are observed, indicating the formation of germanene.^[5]^ In this sample preparation, hBN transfer is added to the all-*in-situ* UHV growth reported in this study. Stiil, the conditions for Ge(111) surface cleaning, Ag thin film deposition, surface cleaning by Ar^+^ ion sputtering, and germanene segregation by annealing are the same.

1. Discussion on the ambient stability performance of germanene on Ag(111)/Ge(111)

Germanene is not stable in the ambient air and oxidizes. Figure S14 shows O 1*s* (Figure S14a) and Ge 3*d* (Figure S14b) SRXPS spectra of germanene on Ag(111)/Ge(111) after O_2_ exposure at various pressures at RT. The results indicated that an O_2_ pressure above ~ 10^-4^ Pa caused severe oxidation to germanene. Since the oxidation here is accelerated by the BA gauges as discussed in Figure S2, the oxidation of germanene by O_2_ gas molecules is much slower. Nevertheless, as indicated by the results of Figure 3a (air-ox.), germanene is unstable in air and eventually undergoes oxidation.

**Table S1.** Obtained fitting parameters of the Ge 3*d* of as-grown germanene on Ag(111)/Ge(111) (Figure S4).

| Content | Value |
| --- | --- |
| Area ratio of Ge 3*d*_3/2_ divided by Ge 3*d*_5/2_ | 0.73 |
| Peak position of Ge 3*d*_3/2_ | 29.02 eV |
| Peak position of Ge 3*d*_5/2_ | 29.59 eV |
| Full width at half maximum | 0.46 eV |
| Ratio of Gaussian | 0.20 |
| Ratio of Lorentz | 0.80 |
| Coefficient of determination (R^2^) | 0.995 |
| Chi-squared | 0.01391 |

**Table S2.** Experimentally observed core level shifts due to surface oxidation states of Ge. The average shift per oxygen atom was derived from these values.

|  | Core level shifts (eV) | | | | Average  (eV) | Ref. |
| --- | --- | --- | --- | --- | --- | --- |
|  | Ge^+^ | Ge^2+^ | Ge^3+^ | Ge^4+^ |  |  |
| Germanene  on Ag(111) | - | 1.5  1.3 | 2.9 | 3.3 | 0.83 | This work |
| Ge  (bulk surface) | 0.8 | 1.8 | 2.6 | 3.4 | 0.85 | ^[1]^ |


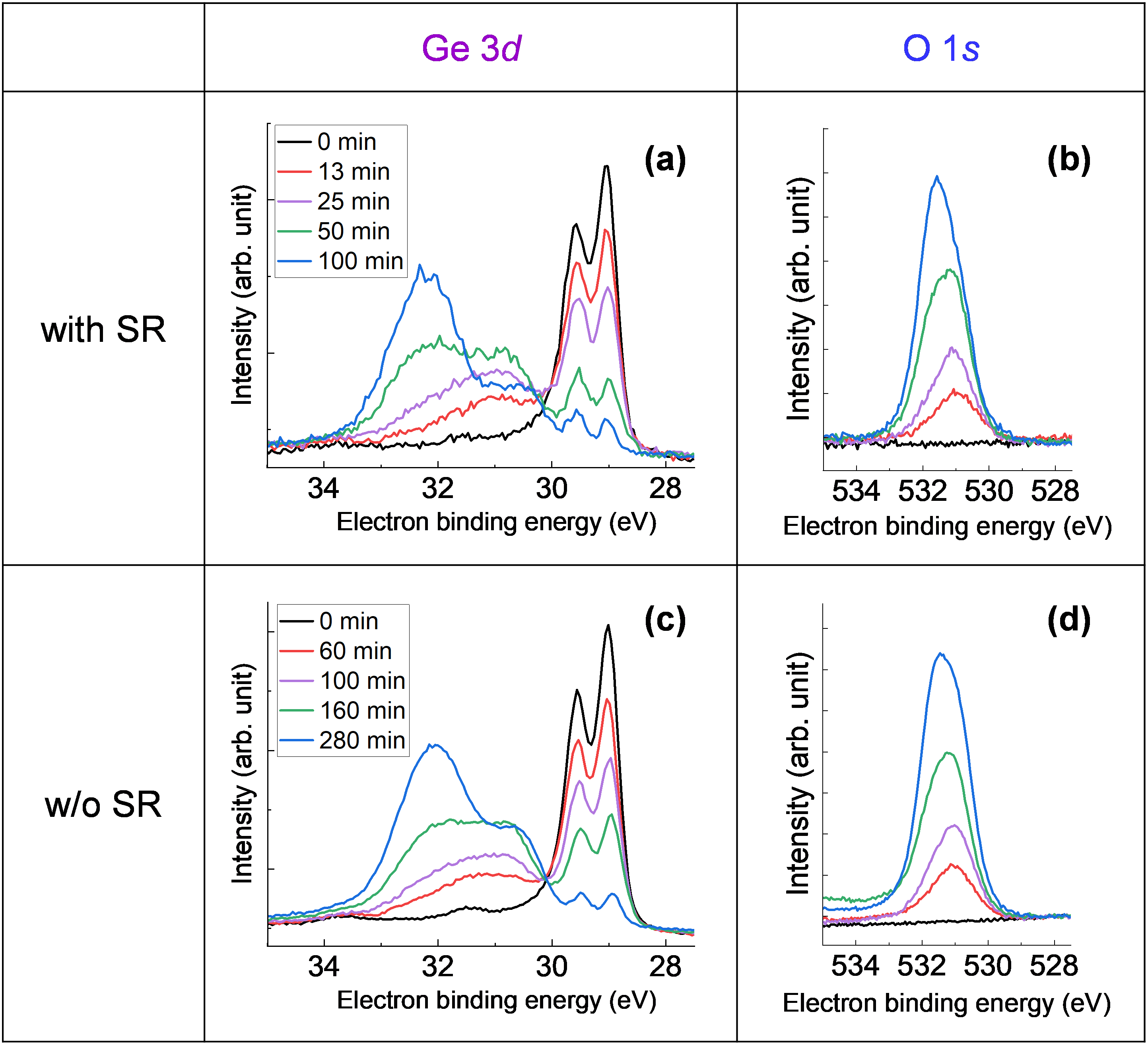


**Figure S1.** Comparison of Ge 3*d* (a,c) and O 1*s* (b,d) spectra of oxidized germanene at different O_2_ exposure times with (a,b) and without (c,d) SR. The two BA gauges remained in use during the O_2_ exposure. The backfilled O_2_ pressure was 2 ×10^-4^ Pa, and its exposure times were 0, 13.3, 25, 50, and 100 min for (a,b) and 0, 60, 100, 160, 280 min for (c,d).


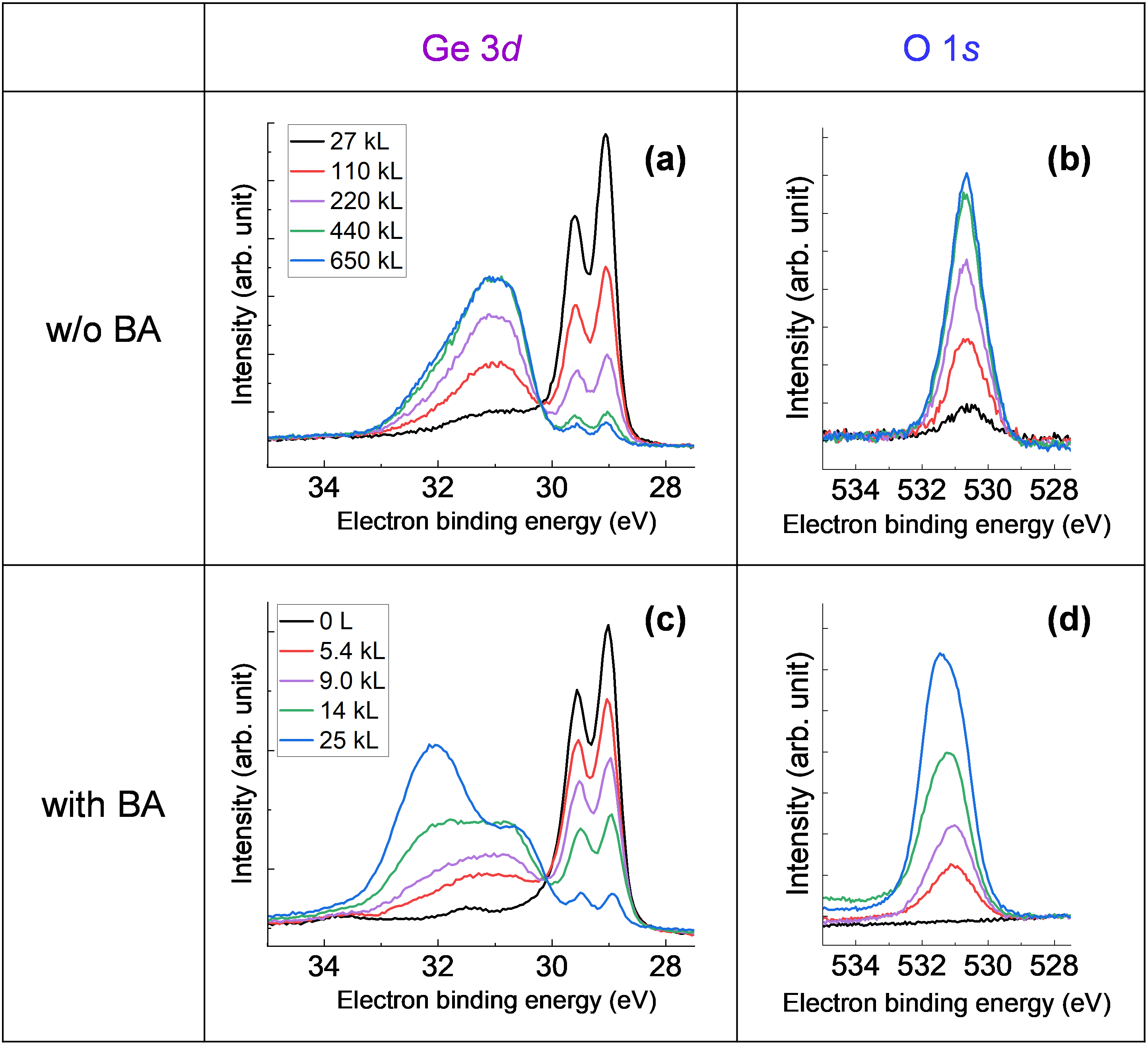


**Figure S2.** Comparison of Ge 3*d* (a,c) and O 1*s* (b,d) spectra of oxidized germanene at different O_2_ exposure dosages with (c,d) and without (a,b) the oxidation acceleration by the two BA gauges. The backfilled O_2_ pressure with BA was 2 ×10^-4^ Pa (c,d), while the backfilled O_2_ pressure w/o BA ranged from 7.6 ×10^-3^ to 3 ×10^-2^ Pa (a,b).


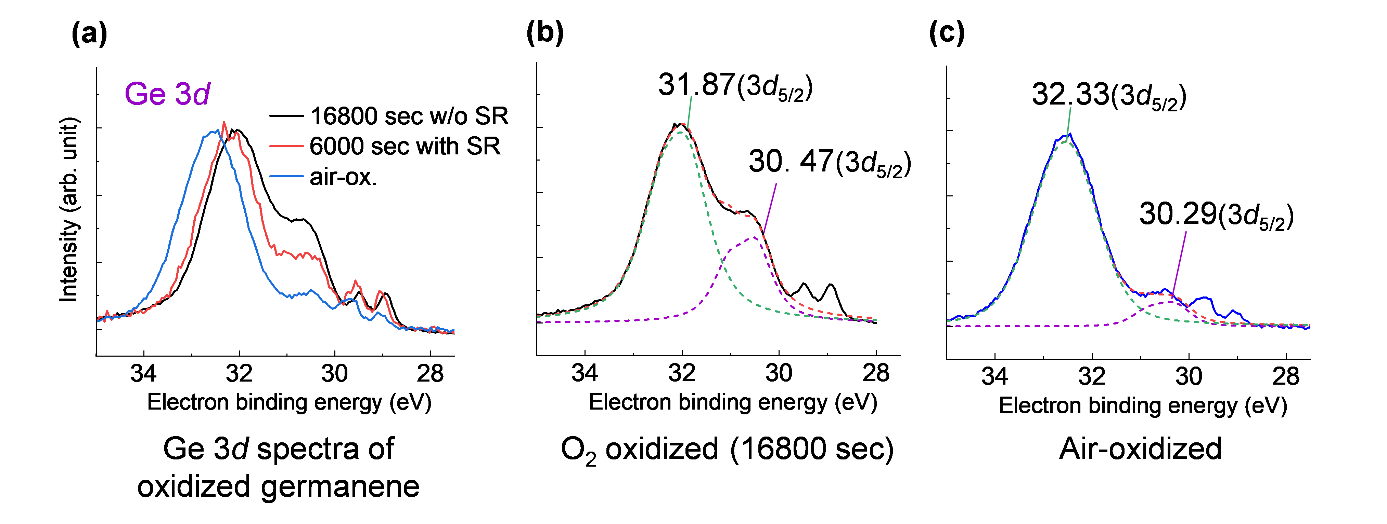


**Figure S3.** Comparison of the oxidation state of various oxidized germanene. (a) Ge 3*d* spectra of oxidized germanene under three different oxidation conditions: O_2_ exposure at 2×10^-4^ Pa for 16800 sec (280 min) under SR irradiation with the two BA gauges, O_2_ exposure at 2×10^-4^ Pa for 6000 sec without SR irradiation with the two BA gauges, and air oxidation for 3 hours. (b,c) The curve-fitting results for dry O_2_-exposed (b) and air-exposed (c) oxidized germanene.


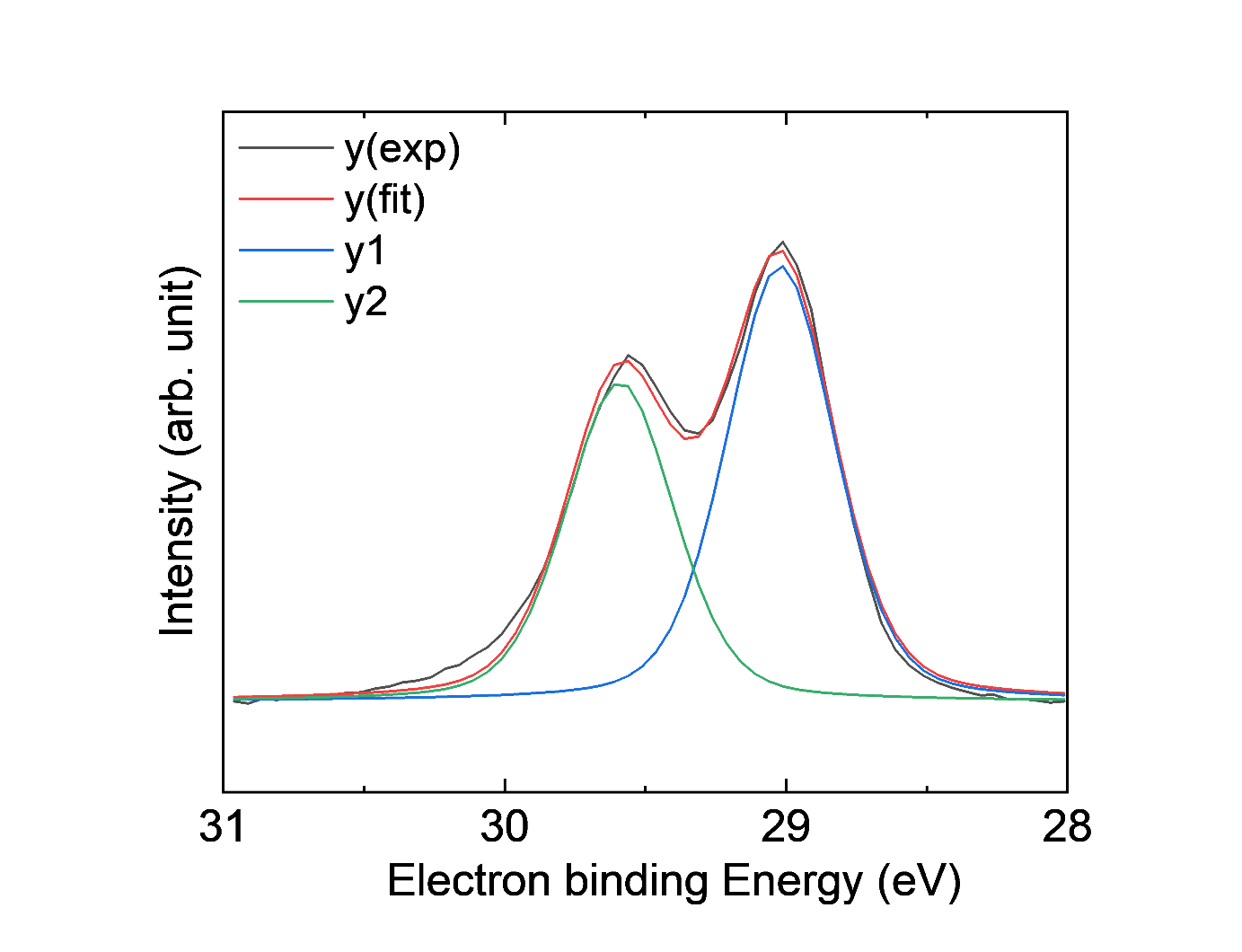


**Figure S4.** Fitting of Ge 3*d* of germanene. The obtained convolution curve (y(fit)) was used as a standard peak for fitting the Ge 3*d* spectra of oxidized germanene, as shown in Figure S3.


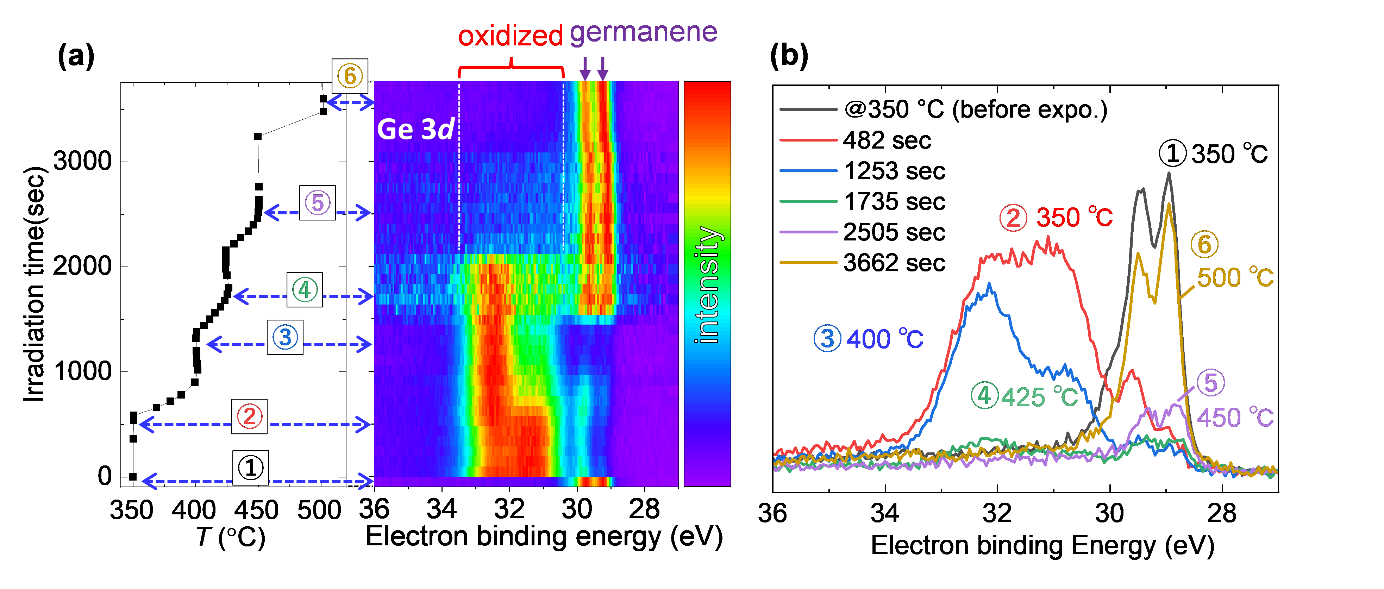


**Figure S5.** *In situ* SRXPS measurement of O_2_ SSMB irradiation of germanene at high temperatures. (a) Temperature transition (left) and color-scaled photoelectron intensity around Ge 3*d* (right) over irradiation time. (b) Ge 3*d* SRXPS spectra at various irradiation times. The approximate temperatures corresponding to each spectrum are displayed. The correspondence between the irradiation time and each spectrum is also shown by the double-sided arrows in (a).


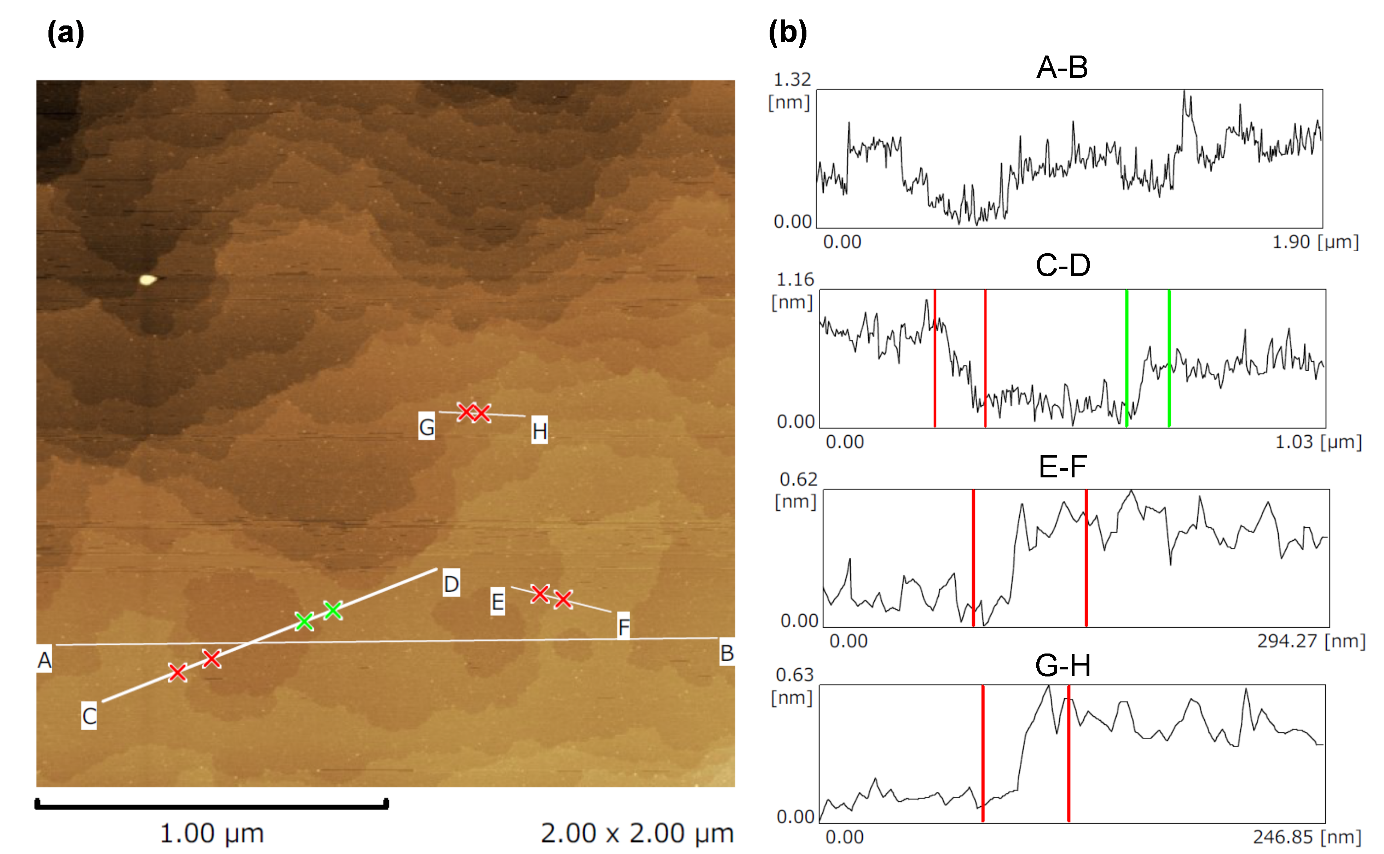


**Figure S6.** AFM observation of Ge(111) surface. Dynamic force mode was used for the observation. (a) Typical AFM topography image of the cleaned Ge(111) surface. (b) Line profiles in (a).


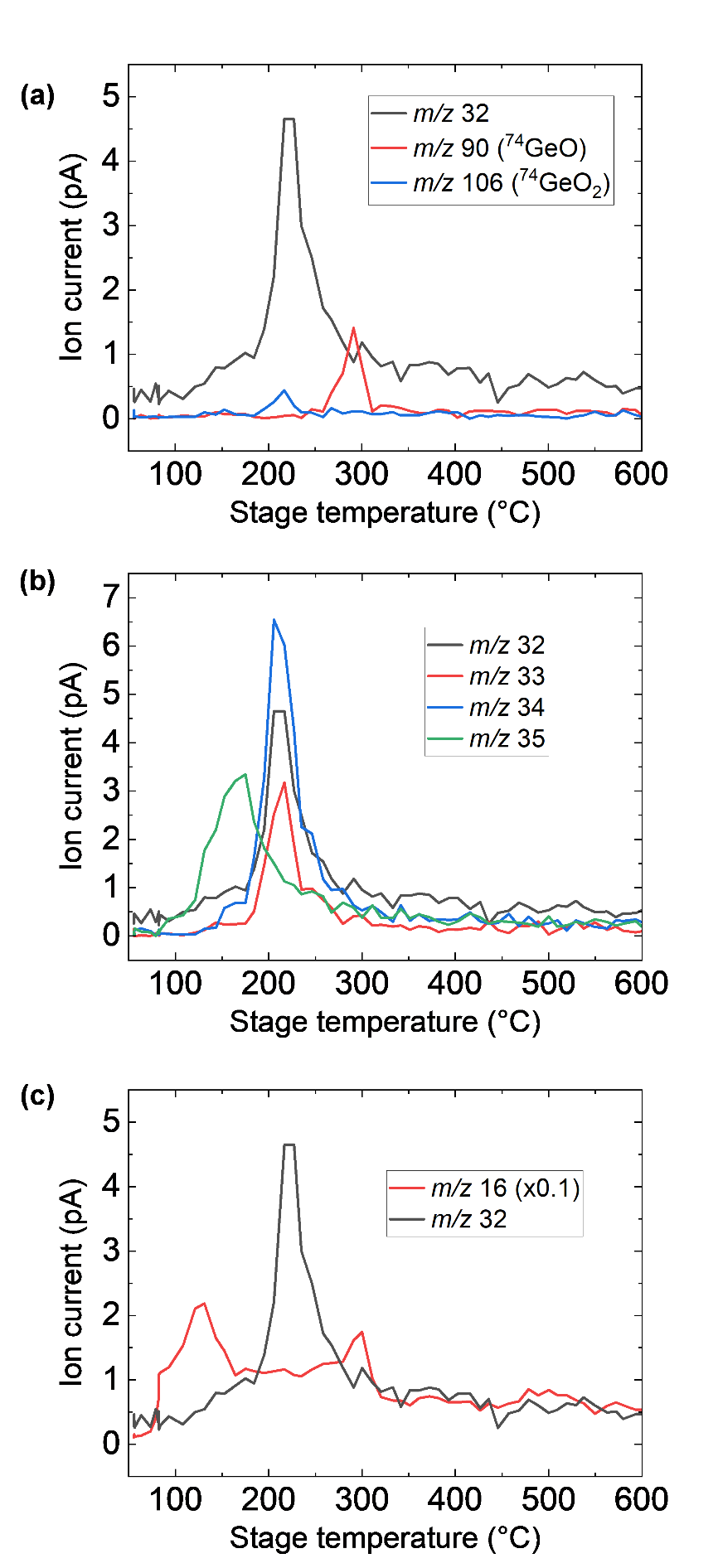


**Figure S7.** Comparison of *ex situ* TDS spectra for the selected *m/z* to identify the desorbed species from annealed oxidized germanene on Ag(111)/Ge(111): (a) TDS spectra at *m/z* 32, 90 (^74^GeO), and 106 (^74^GeO_2_); (b) TDS spectra at *m/z* from 32 to 35; (c) TDS spectra at *m/z* 16 and 32. The ion currents for TDS at *m/z* 16 in (c) are multiplied by 0.1 for easy comparison.


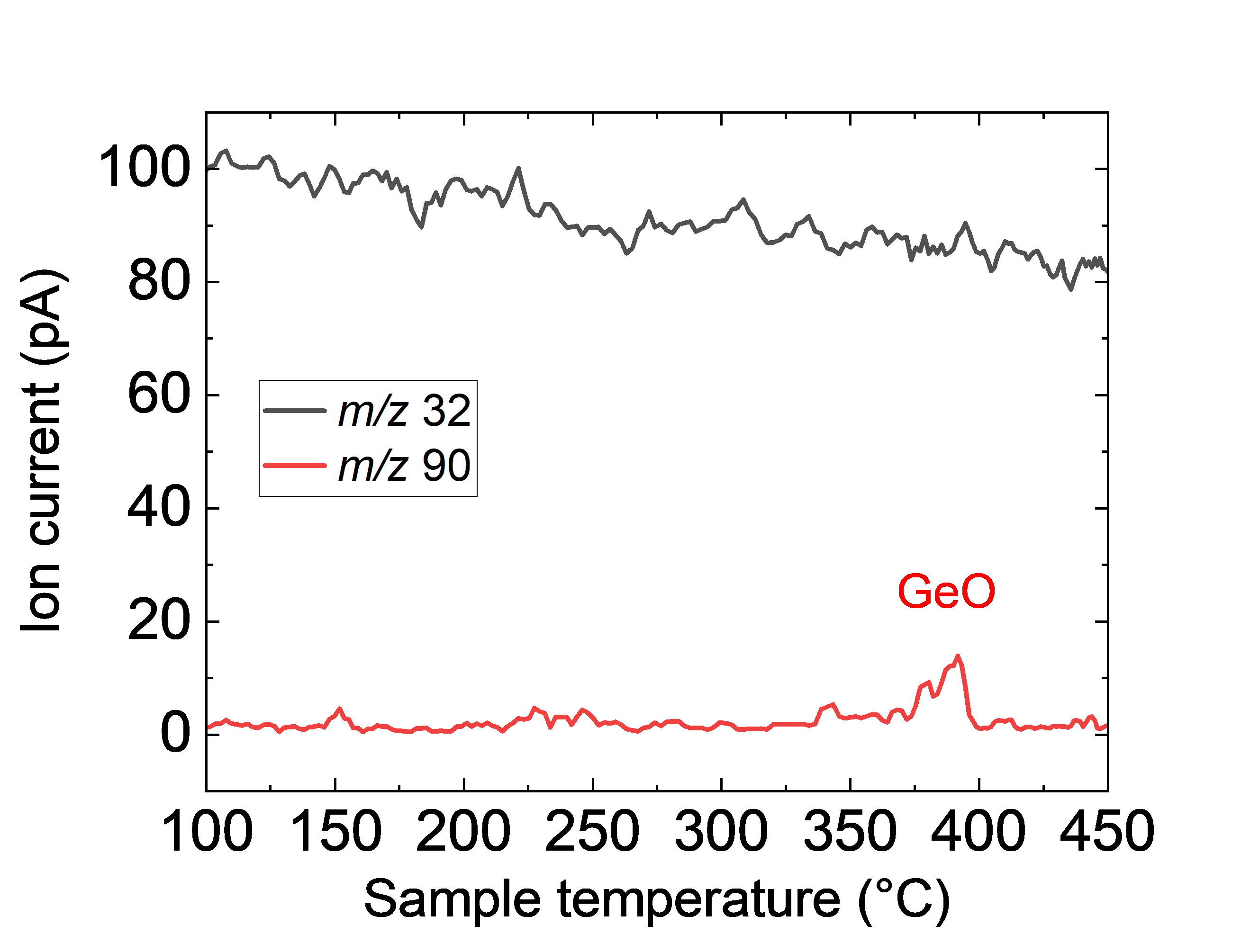


**Figure S8.** *In situ* TDS of oxidized germanene on Ag(111)/Ge(111) at *m/z* 32 and 90.

**Figure S9.** Ge 3*d* SRXPS spectra of oxidized germanene at RT after heating at different temperatures.

**
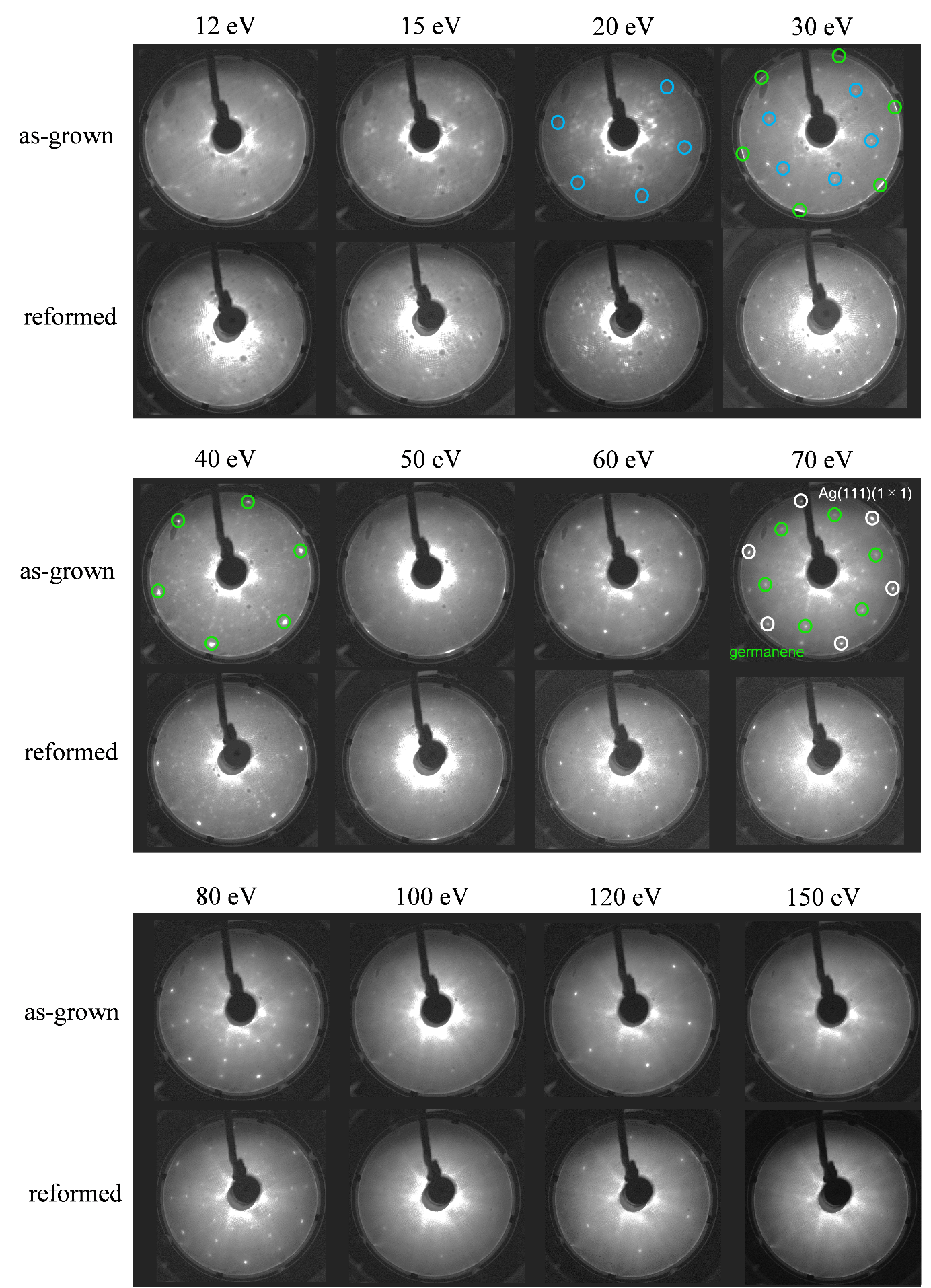
**

**Figure S10.** LEED patterns for different acceleration electron energies of as-grown and reformed germanene

**
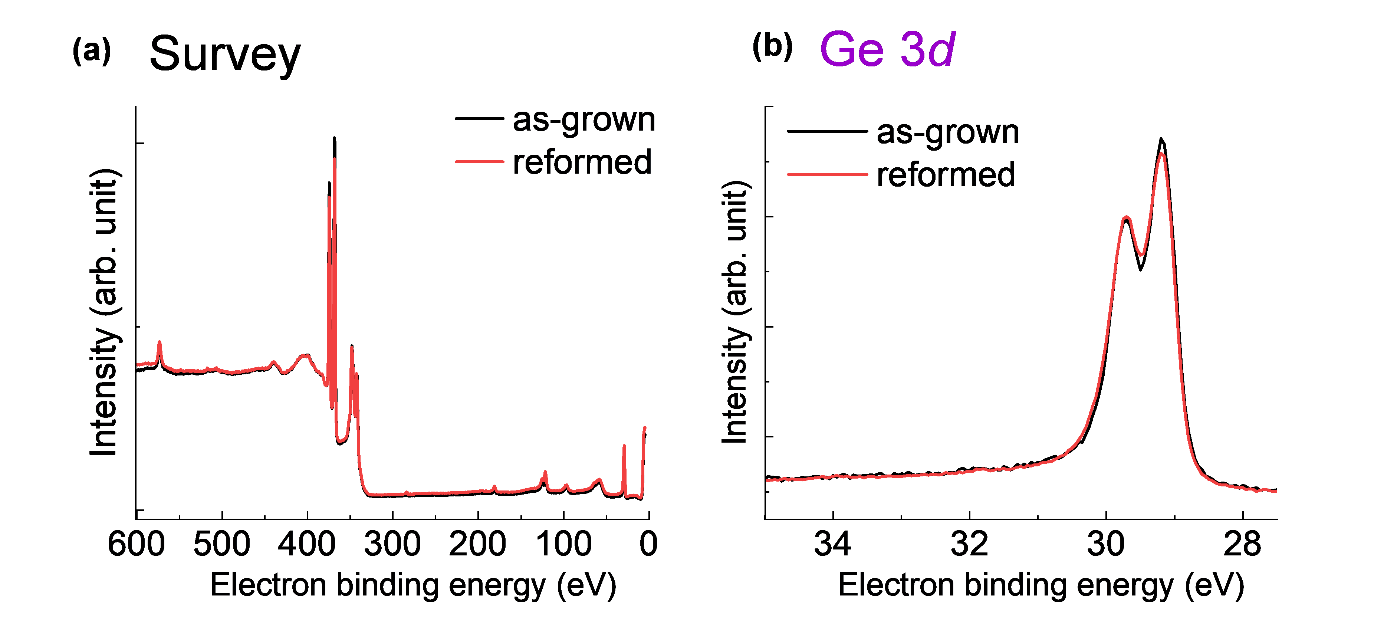
Figure S11.** (a) Survey and (b) Ge 3*d* SRXPS spectra of as-grown and reformed germanene.


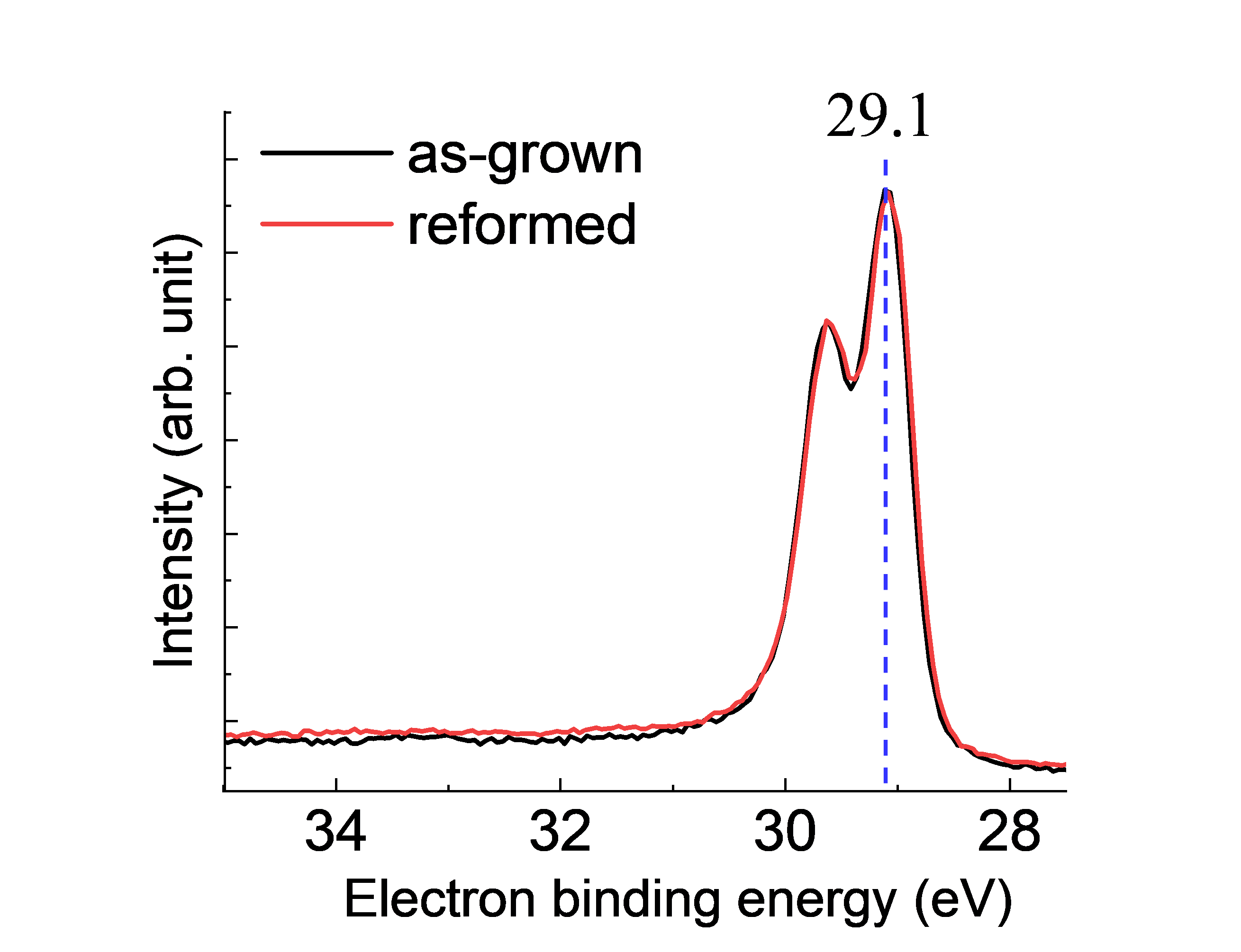


**Figure S12.** Ge 3*d* SRXPS spectra of as-grown and reformed germanene with the photoelectron detection angle of 70 degrees.


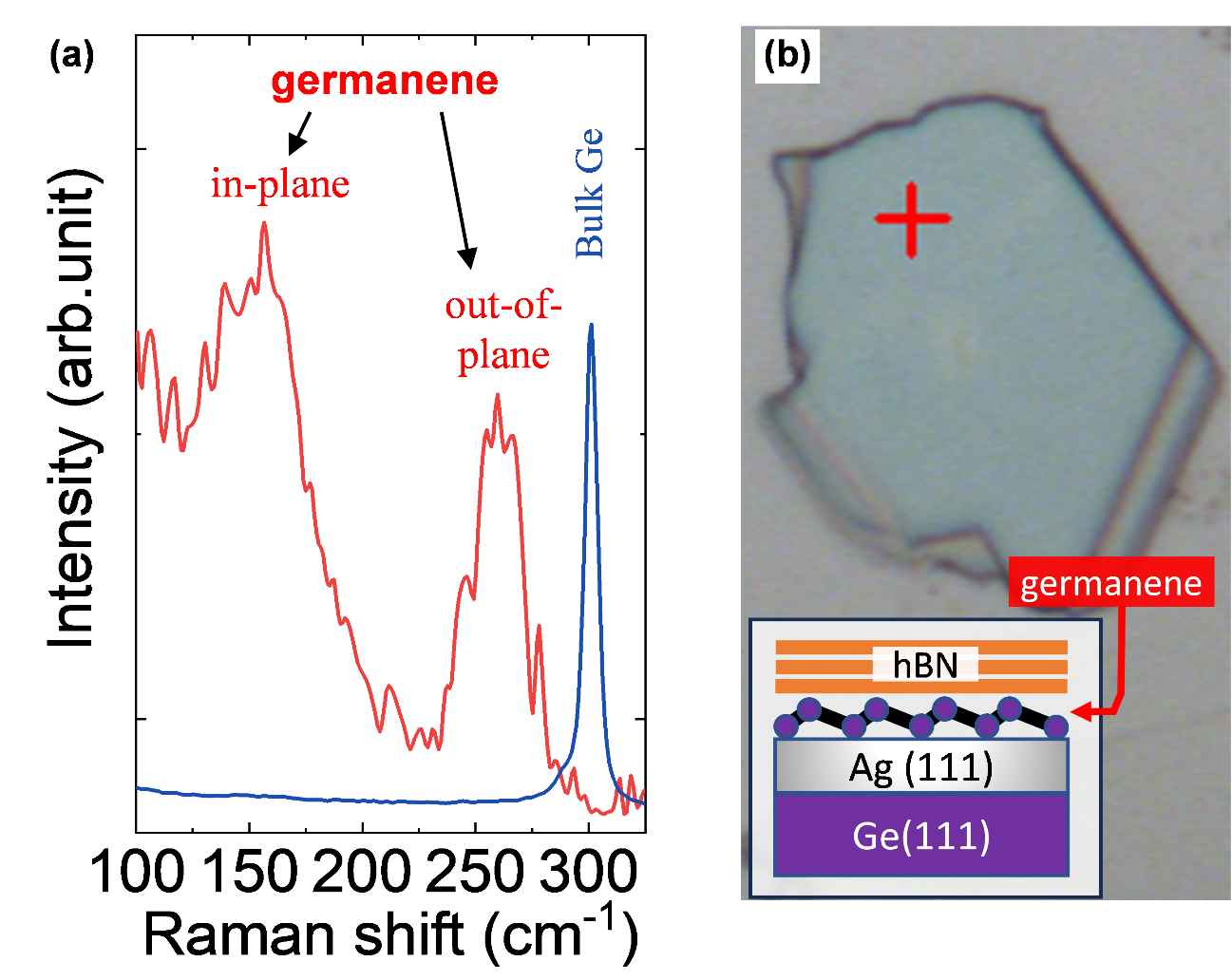


**Figure S13.** (a) Raman spectra of hBN-capped germanene on Ag(111)/Ge(111) and bulk Ge. In-plane and out-of-plane vibrational modes of germanene are observed. (b) Optical microscope image of hBN/germanene/Ag(111)/Ge(111) and cross-sectional schematic.


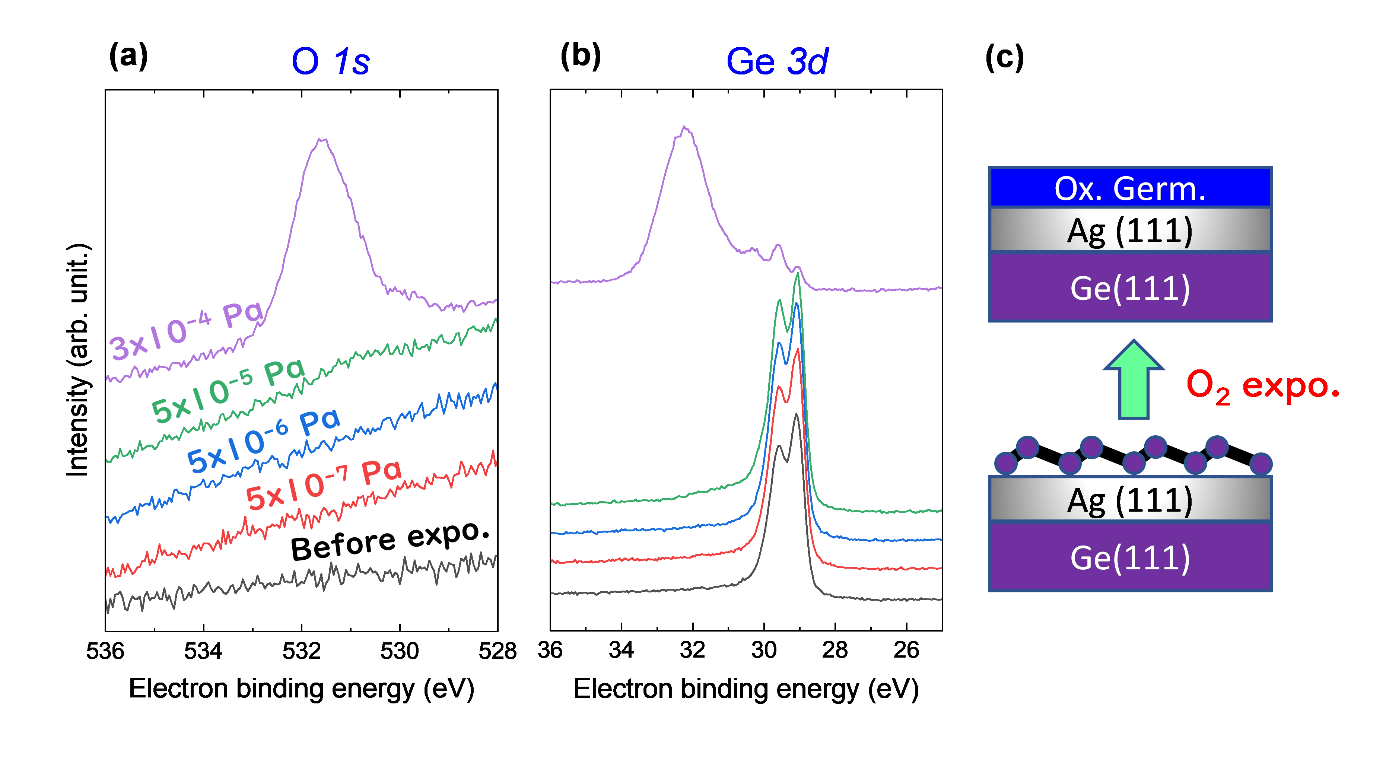


**Figure S14.** (a) O 1*s* and (b) Ge 3*d* SRXPS spectra of germanene on Ag(111)/Ge(111) after O_2_ exposure at various pressures at RT. (c) Schematic of the sample. The O_2_ exposure was performed with the following backfilled O_2_ pressure and exposure time: 5 × 10^-7^ Pa for 30 min, 5 × 10^-6^ Pa for 30 min, 5 × 10^-5^ Pa for 60 min, and 3 × 10^-4^ Pa for 105 min. Note that the oxidation by the backfilling O_2_ here is accelerated by the BA gauges discussed in Figure S2.

References

[1] D. Schmeisser, R. D. Schnell, A. Bogen, F. J. Himpsel, D. Rieger, G. Landgren, J. F. Morar, *Surf. Sci.* **1986**, 172, 455.

[2] S. Wang, H. Liu, T. Nishimura, K. Nagashio, K. Kita, A. Toriumi, *ECS Trans.* **2013**, 50, 557.

[3] J. Franey, G. Kammlott, T. Graedel, *Corros. Sci.* **1985**, 25, 133.

[4] J. Yuhara, H. Shimazu, K. Ito, A. Ohta, M. Araidai, M. Kurosawa, M. Nakatake, G. Le Lay, *ACS Nano* **2018**, 12, 11632.

[5] S. Suzuki, T. Iwasaki, K. K. H. De Silva, S. Suehara, K. Watanabe, T. Taniguchi, S. Moriyama, M. Yoshimura, T. Aizawa, T. Nakayama, *Adv. Funct. Mater.* **2021**, 31, 2007038.
